# Weaving social networks from cultural similarities on the neolithisation process in the Western Mediterranean: Evolutionary trajectories using projectile tools

**DOI:** 10.1371/journal.pone.0306027

**Published:** 2024-07-30

**Authors:** María Barrera-Cruz, Oreto García-Puchol, Joaquín Jiménez-Puerto, Alfredo Cortell-Nicolau, Joan Bernabeu-Aubán

**Affiliations:** 1 PREMEDOC Research Group, Departament de Prehistòria, Arqueologia i Història Antiga, Universitat de València, València, Spain; 2 MacDonald Institute for Archaeological Research, Department of Archaeology, University of Cambridge, Cambridge, United Kingdom; University of California Santa Cruz, UNITED STATES

## Abstract

In this paper, we concentrate on the neolithisation process in Mediterranean Iberia through a diachronic view (from 8600–6800 cal. BP), focusing on social interaction as a factor in articulating new cultural ties. To do this, we apply techniques centred on similarities in material culture by applying Social Network Analysis (SNA). For the first time, we point to the geometric projectiles, taking into account their recurrence in both Mesolithic and Neolithic groups as part of their characteristic hunting equipment. We hypothesise that patterns of cultural variability would express the changing flow of information between communities according to their mobility strategies (last hunter-gatherer groups), including economic and social behaviour, and that these relationships will be restructured with the arrival of the newcomer farmers and herders and their new spatial and social arrangement. The results obtained allow us to describe a connected and homogeneous Late Mesolithic network dramatically structured by the Neolithic arrival. Since then, a heterogenous pattern emerged, involving connected periods, network ruptures, and small-world phenomena. The emergence of this characteristic could support the flow of information when the network presents a clustered structure, the last probably due to regionalisation events. These diachronic dynamics fit well with demographic and socioecological trends observed from regional literature.

## 1. Introduction

The spread of farming and herding practices from the Fertile Crescent region in Western Asia to Europe stands out as one of the most important irruptive episodes affecting recent past human social dynamics. This effect is also visible at different scales and times along the world coinciding with the appearance of domestic plants and animals. New demographic patterns triggered by changes in human-based diet, settlement organisation, and social relationships, largely transformed human ecosystems compared with previous hunter-gatherer societies. Among them, the increasing population and social relationships constitute crucial aspects for fostering migratory movements [1–4]. If we focus on the Western Mediterranean, the Neolithic transition can be explained as a demic and cultural model involving the pioneering arrival of herders and farmers in the first half of the VIII millennium cal. BP, which subsequently, promoted potential contact scenarios with the last Mesolithic population [5–11]. Recent published ancient DNA results underscore these interbreeding signals [12, 13]. Population expansion along the Mediterranean corridor spreads the “Neolithic package” including a new cultural background that could interplay with cultural patterns present in the last Mesolithic incumbent populations. Pointing on a diachronic view, our aim consists of characterizing patterns of cultural change and connectivity networks encompassing the last Mesolithic and the first Neolithic episodes in the Iberian Mediterranean to solve evolutionary questions relating to information flow and cultural linking.

The similarities of cultural change patterns regarding biological evolutionary processes are one of the key epistemological foundations for analyzing spatial and temporal cultural transmission processes [14, 15]. To do this, it is essential to order cultural sequences and recognise evolutionary cultural patterns.

Social connectivity in past human societies has been explored since a long time ago through structural and agency approaches relying on cultural similarities and material flow over space and time [16, 17]. The last developments of Complexity theory, and the subsequent analysis of human societies as complex systems characterised by non-linear dynamics, that result in emergent phenomena, have triggered the focus on exploring cultural material links for explaining local and regional social interactions considering multi-scalar approaches [18, 19]. Among them, Social Network Analysis (SNA) constitutes a paradigm for studying social connectivity involving individual agents (actors or nodes) and the structural organisation of communities by implementing methods applied successfully in other social and physical sciences [17, 20]. Briefly, a network or graph consists of a group of nodes (again, individual agents or actors) joined in pairs by lines or branches (edges) that define the connection between them [21]. Considering this approach, we look at social relationships among communities based on the premise of the important role played by horizontal transmission processes produced when information flows between coexisting communities. In this sense, focusing on how information (techniques, styles, beliefs, etc.) or materials (raw materials, objects) are distributed due to social connectivity is crucial to understanding patterns of social dynamics. Both, structure and node analysis, are relevant for investigating the structural analysis (graph level: referred to the general structure emerged) and individual positions within the network (node level: relating to the role of the different agents or actors) [20, 22]. Additionally, the detection of the small world phenomena (when some node facilitates the flow of information also known as “the six degrees of separation” from Milgram’s experiment [20, 23], social structures based on power law distributions (when some hubs centralise the circulation), or stability or fragmentation periods between social structural dynamics, among others, could be recognised through the combination of several algorithms and statistical metrics.

In this paper, based on SNA, we concentrate on the neolithisation process in Mediterranean Iberia through a diachronic view that incorporates Mesolithic/Neolithic interaction as a factor to articulate new cultural connectivity patterns. We posit that cultural diversity patterns may reflect the evolving transmission of information among communities based on their mobility strategies and economic and social behaviours. In this sense, we anticipated restructuring of these dynamics, along with their novel spatial and social configurations, due to the arrival of newcomer farmers and herders. Accordingly, this research is mainly pointed to (1) the spatial structure of social relations among nodes pointing to individual and general levels, (2) the diachronic dynamics of social networks considering both Mesolithic and Neolithic societies, (3) the emergent phenomena that could be approached considering several diagnostic analyses (small world phenomenon), and (4) the comparison of the patterns obtained results with evolutionary dynamics including branching and blending effects in cultural change.

**Fig 1:**
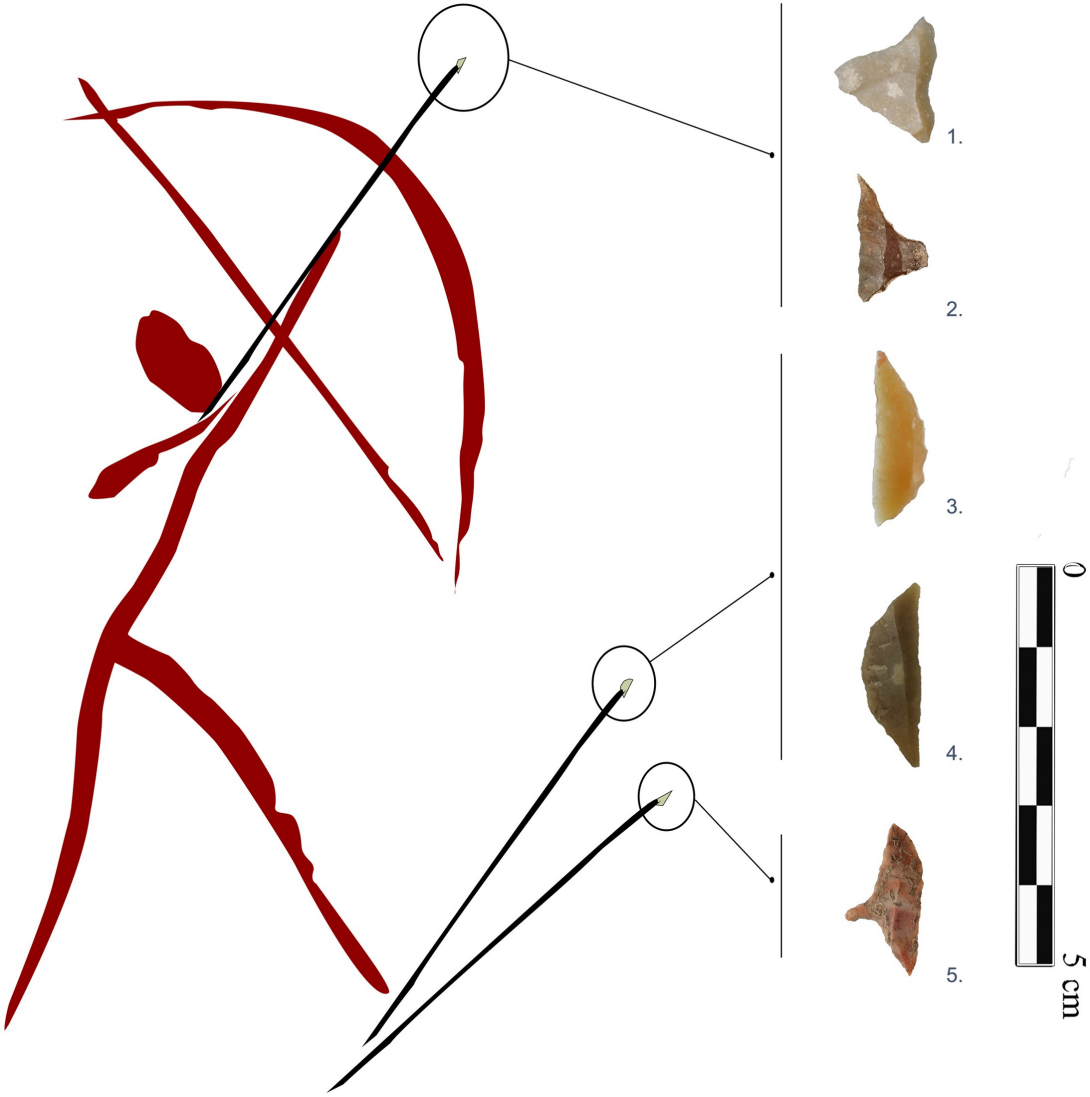
Some examples of the geometric microliths in the sites considered for this study. From top to bottom, the geometrics belong to the sites of Mas d’Is (1, 3), Cueva de la Cocina (2, 5), and Cova de l’Or (4). Information about the total sample used is available in Table 1 and S1 File. In this figure, images of microliths 1, 2 and 5 are published for the first time in this work; geometric 3 is republished from [24] under CC BY license, with permission from A. Cortell-Nicolau and O. García-Puchol, original copyright 2020; and geometric 4 is republished from [25], under CC BY license, with permission from A. Cortell-Nicolau and O. García-Puchol, original copyright 2021. The illustration was entirely designed and elaborated by the authors of this work.

We focus on the geometric projectiles (Fig 1) taking into account that this is a recurrent object in both Mesolithic and Neolithic groups as part of their characteristic hunting equipment. The basic concern consists of the premise that the analysis of material culture considering the variability of artefact types between nodes reveals information of some level of connectivity among them as a way to explore affiliation networks [20]. Consequently, artefact occurrence transformed as a matrix of similarities among nodes (archaeological level by site) allows us to understand patterns of social proximity. We evaluate variability in projectile tools through a “paradigmatic” traits-based classification based on cultural similarities from high-resolution temporal windows of 200 years. To build them we have designed an accurate filter of archaeological contexts and radiocarbon dates (from circa 8600 to 6800 cal. BP).

### 1.1 Western Mediterranean Iberia neolithisation background

The Western Mediterranean Iberia takes part of the evolutionary dynamics of the last hunter-gatherers and the first farmers described in a wide Mediterranean view [26]. Holocene paleoenvironmental changes refer to particularities at a regional and microregional scale. However, regional conditions encapsulate some general effects such as the rise of temperature and humidity that allowed forest progression, and the progressive sea level increase that modified coastal territories [27–29]. Even though regional research refers to distinct Mesolithic cultural dynamics, in a general perspective it is possible to determine some confluences that can be summarised at least by two great technocomplexes: the First Mesolithic, which includes several industries where the importance of expedient flakes knapping techniques is pivotal [30, 31], and the blades and trapezes technocomplex (also named Second Mesolithic) that reveals new lithic parameters based on blade technology and geometric projectiles [32, 33]. In the Mediterranean Iberia, the oldest technocomplex, named (Notches and Denticulated Mesolithic -NDM-, extends from circa 10.200 to 8600 cal. BP, while the second (Geometric Mesolithic -GM-) starts from circa 8700 to 7500 cal. BP [34, 35]. With respect to the Geometric Mesolithic, despite this general definition, current data exhibit spatial variation expressed by some regional information vacuums -as the absence described in Catalonia [36], its not well-defined presence in Andalusia [37]-, or its variability when we point to the entire Iberian Peninsula [38–40]. Effectively, the main confluence showed by lithic industries corresponds with the Mediterranean corridor (eastern Iberia), the Ebro valley, and the Portuguese coastal and estuarine areas, mainly the Sado and Tagus rivers [41]. A morphometric evolution is visible regarding geometric projectile tools where an initial predominance of asymmetric trapezoidal shapes is followed by the abundance of triangles with concave edges (also known as “Cocina type” and “Muge type”) [42, 43].

The first neolithic contexts in Iberia are recognized in a wide area around 7600 cal BP including Catalonia, Ebro Valley, Valencia region and Southern Andalusia [25, 26, 44, 45]. Recent investigations have shown the presence of early *impressa* contexts in a few numbers of archaeological sites located in the Cap de la Nau/Serpis Valley -Valencia region- [45, 46], maybe as the first traces of a pioneering arrival at the Mediterranean Iberian coast following a leapfrog movement [6]. Nevertheless, the *cardial* impressed ware constitutes the best early extended pottery tradition followed by a regionalisation marked by the generalisation of other decorative pattern styles from the end of the VIII millennium cal. BP [44]. The newcomer farmers and herders extend along the diverse territories adapting these new economic patterns to the ecological conditions in an arrhythmic process involving some fast trajectories (coastal areas and Ebro valley), in which interaction with foragers seems to be more rapidly blurred ([35]; see also [47] for Portugal). The new cultural package incorporates pottery, polished stone tools, and a renewed toolkit that integrates, among others, glossed blades, bifacial drills, and also, geometric projectiles including renewed shapes and retouch techniques [25, 42, 48]. As mentioned before, here we analyze in deep cultural variability dynamics of geometric projectiles present in a *longue durée* perspective among Mesolithic and Neolithic groups.

## 2. Materials and methods

This section presents the data employed in our study, clarifying the methods used for data acquisition, and organization. Subsequently, a comprehensive view of the procedures involved in network construction and analysis is provided.

Data was collected from primary and bibliographic sources (see details in Table 1). Analysis was developed using R statistical language [49] and Gephi software [50] (details specified below). Script and data are included in the supplementary material to promote a fully reproducible study.

**Table 1:** The number of geometrics present in every site and level. In the column ’n’, the total number of pieces included at the beginning of this study.

Site	Level	Time frame	n	appearance > 1^a^
Artusia	III	8600–8401	4	4
	V	8200–7801	2	2
Abric de la Falguera	VIII	8400–8001	2	2
	Xa	8400–8201	1	1
Abric del Xicotó	II	7200–7001	4	2
Ángel 2	2a2	8000–7801	12	12
Atxoste	IV	8200–7601	62	60
	IIIb2	7600–7401	22	21
Barranquet	79	7600–7401	2	2
Benàmer	I	8400–8201	32	32
	II	7600–7401	3	2
Botiquería dels Moros	2	8600–8201	43	42
	4	7800–7601	16	12
Cabezo de la Cruz	Cabaña	8000–7801	8	8
Casa Corona	LM	8000–7801	12	11
Caserna de Sant Pau	IV-base	7400–7201	1	1
Castillejos	I	7400–6801	2	2
Can Sadurní	18 (IIIC)	7400–7200	1	1
Cingle de Mas Cremat	VI	7800–7401	1	1
	V	7800–7601	8	8
	III	7000–6801	1	1
Costamar	NII	7000–6801	12	10
Cueva de Cocina	A1	8600–8201	57	57
	A2	8400–8001	85	85
	B1	8000–7601	96	96
	B2		173	173
	B3	7800–7401	20	20
Cova de l’Or	IV	7400–7001	11	10
	V	7400–7201	27	26
	VI	7400–7001	44	43
Cova de les Cendres	XI	7600–7201	3	2
	X	7400–7001	1	0
	IX	7200–7001	2	2
	VIIa	7200–6801	1	1
Cova dels Trocs	53	7000–6800	2	2
Coves del Fem	103	7600–7401	4	2
Cueva de Chaves	Ib	7600–7201	38	23
	Ia	7200–7001	6	4
Cueva de Nerja	NM10	7400–7201	5	4
	NV2	7200–7001	1	1
Cueva del Toro	IV	7200–7001	4	4
El Abrigo de Valcervera	b	8000–7801	4	4
El Collado	I-3	8600–8401	4	4
El Esplugón	3-inf	8000–7601	47	45
Espantalobos	c	8400–8201	7	7
Forcas II	II	8000–7801	14	14
	IV		26	24
La Draga	ABC	7200–6801	14	9
Les Guixeres de Vilobí	A	7600–7401	5	3
	B	7000–6801	1	1
Mas d’Is	VIb	7600–7401	2	1
Mas Nou	III (Burial)	7800–7600	2	2
Pontet	e	8200–8001	14	14
Valmayor	XI-III	7000–6801	5	4
Total			976	924

^a^Column ’appearance > 1’ refers to microliths whose typology appears on more than one occasion. Details about the data source are available in the S1 File.

### 2.1 The sample

Given that one of the main focal points of our work is to describe dynamics in cultural variability from a high-resolution record, this study did not consider samples from mixed, merged, or not-dated contexts. Instead, before including any geometric projectile in the sample, we verified the integrity of the stratigraphic information and the association between that context, the geometric, and the radiocarbon data available. We have collected projectile data from direct analysis (assemblages studied by us), and also from bibliography. Also, since we need to classify the assemblage from images uniformly, we discard the geometrics with poorly drawn information available.

We always linked the material cultural data with available radiocarbon dates to organise assemblages in chronologically consistent samples according to the established criteria (See S1 File). For the selection of radiocarbon data, (1) samples with a standard deviation greater than 100 years were discarded, (2) short-lived dates were selected over long-lived ones, (3) aggregate samples were discarded, and (4) we prioritised radiocarbon data from the layer or unit where the geometric was precisely located. If this was impossible, we selected radiocarbon dates from units or layers culturally consistent with the original unit where the geometric was coming from (excluding clear palimpsests).

**Fig 2:**
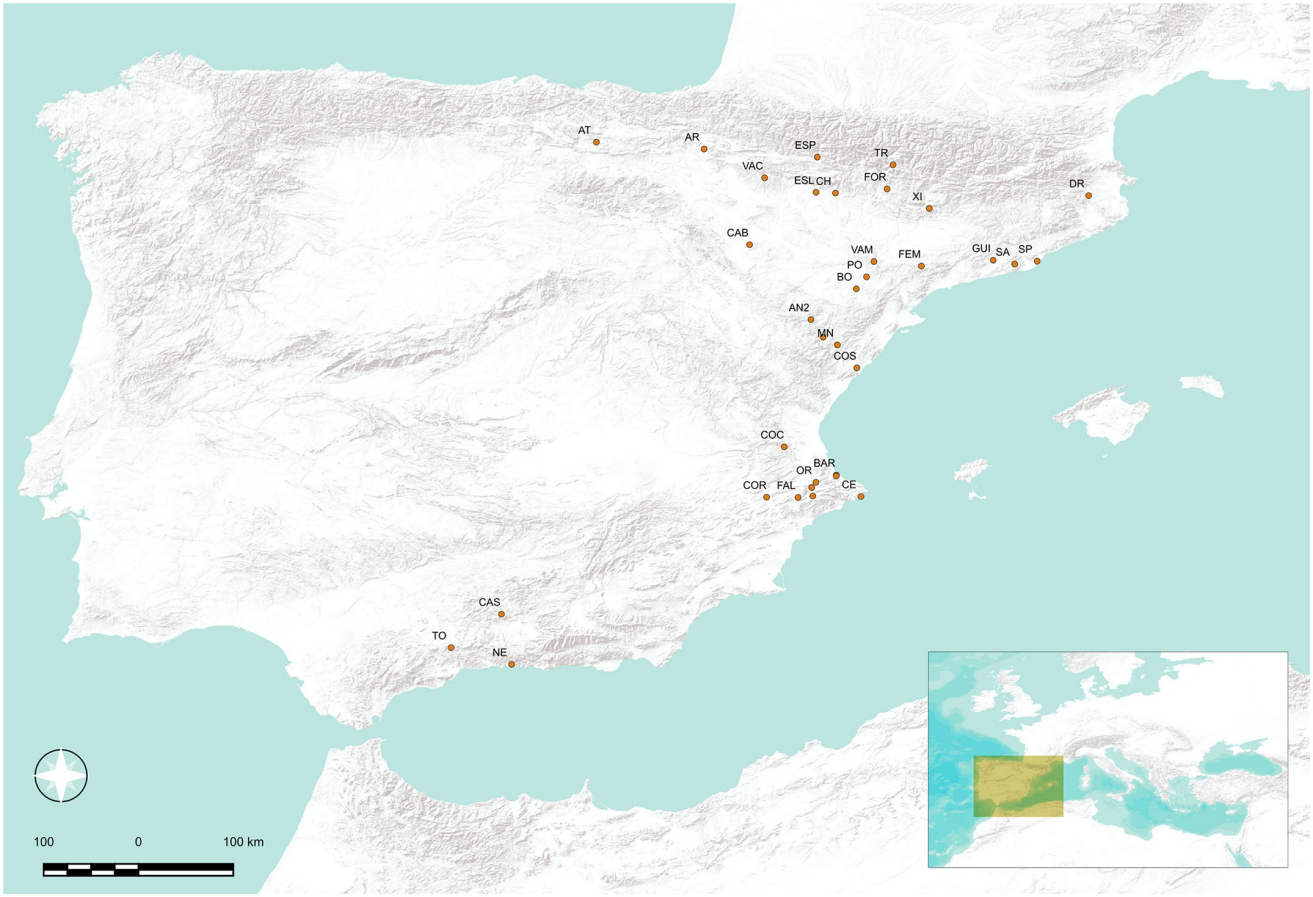
Sites considered for this study. Only the sites containing geometric microliths for the timeframe under study are shown. Sites: AN2 (Ángel2), AR (Artusia), AT (Atxoste), BAR (Barranquet), BE (Benámer), BO (Botiqueria dels Moros), CAB (Cabezo de la Cruz), CAS (Castillejos), CE (Cendres), CH (Chaves), COC (Cocina), COL (Collado), COR (Casa Corona), CRE (Mas Cremat), DR (Draga), ESL (Espantalobos), ESP (Esplugón), FAL (Falguera), FEM (Coves del Fem), FOR (Forcas II), GUI (Guixeres), MIS (Mas d’Is), MN (Mas Nou), NE (Nerja), OR (Cova de l’Or), PO (Pontet), SA (Sadurní), SP (Sant Pau), TO (Toro), TR (Trocs), VAC (Valcervera), VAM (Valmayor), XI (Xicotó). Maps modified from ESRI World Terrain Base Map, licensed under the Esri Master License Agreement.

As a result, the database contains 976 geometric projectiles on 54 levels from 34 archaeological sites (see Table 1 for data description and source details). The sample covers 1600 years of Mesolithic and Neolithic occupation over the Mediterranean Iberian Peninsula, including, Northeast, Ebro Valley, East, and Southeast (Fig 2). The boundary selection corresponds with the main chrono-cultural technocomplexes established in Mediterranean Iberia (including the Ebro corridor as a natural edge to promote interregional connectivity) related to the last Mesolithic (Geometric Mesolithic) and Early Neolithic (Early Impressa, Cardial and Epicardial/Postcardial horizons). This work discusses the validity of the archaeological contexts incorporated in the sample in the supplementary material (S1 File). The projectiles used as cultural proxies are consequently associated with the minimal temporal archaeological unit (archaeological level/structure) with radiocarbon dates.

### 2.2 Cultural classification and filters applied in the geometric data

We first classified the projectiles according to a homogeny criterion to construct the adjacency matrices from the microlith assemblages. The analysis of cultural information can proceed in various ways within archaeological contexts.

Conventionally, diverse methodologies have employed typological classifications to categorise and organise the archaeological data. In the case of geometric microliths, the main classification made and used in the Mediterranean Iberia framework is proposed by Fortea [51], who classified the microliths into three main groups according to their main shape (trapezes, triangles and segments) and 18 subgroups. However, this way of categorising archaeological assemblages came under significant criticism in the 1970s and 1980s, highlighting the potential issues associated with a somewhat arbitrary classification of archaeological types [52]. In any case, they are frequently used for practical purposes, such as offering a comparable classification of assemblages among sites.

The above criticisms led to the subsequent development and application of alternative study approaches. To summarise, one could classify these new approaches into two broad groups according to the way they manage the study of cultural traits: (A) On the assumption that stylistic traits alone can explain cultural variance [53], the first of these alternative approaches analyses traits separately (i.e., when studying geometric variability, these approaches would analyse traits such as shape, size, symmetry, or retouch at single-trait level), thereby also avoiding constructions and segregations that may fall into the subjectivity of the researcher [25]. This approach does not assume any relationship between the traits regarding how they are transmitted or how possible selection phenomena operate on them. It is understood, therefore, that each trait can change independently. (B) The other approach operates by constructing paradigmatic types, which produce types without assuming any hierarchy among the traits [54, 55]. This approach does consider the relationship between the traits or variables that define a type or style and, therefore, also understands that, in general, they can be replicated as a whole. Here, we do not rule out the possibility that some traits within a typology may undergo modifications, thereby altering the internal variability of material culture.

In this study, we employ the paradigmatic approach (B) obtained from the sum of traits to calculate cultural distance because we assume that two contexts that share the presence of types related to the same description (sum of traits) will be more similar to each other than those that share isolated traits. This increased similarity may be indicative of a more significant level of connectivity. We have assumed that geometrics constitute a specific category based on the result of several functional analysis that conclude their primary use as projectiles [56–58], thus not considering some issues open for discussion, such as the constraint that the hafting mode of the projectiles may pose.

The traits that defined types were (i) shape, as a simple definition of the geometric shape, thus including trapezoids (Tra), triangles (Tri), and segments (Seg); (ii) symmetry, which defines whether a geometric is symmetric (S) or not (As); (iii) edges form, which defines the condition of the sides, whether these are straight (R), concave (Cc), convex (Cv), or combined (RCc, RCv or CcCv); (iv) retouch, with information about the inclination or mode including retouches abrupt (A) simple (S) or a combination (AS); and (v) direction of the retouch, in this case with the categories of direct (D), alternate (Al), bifacial (B), double bevel (Db) and inverse (I). As a result, we get a total of 93 different types, but given that we are interested in shared types, we filter the data, saving only those types that appear in more than one occasion. In consequence, the final data contains a total of 924 microliths classified into 40 types.

### 2.3 Adjacency matrices

To assign each assemblage to one or another time-bin, we first calibrated all the collected and filtered dates using the IntCal20 calibration curve [59] using the R packages Rcarbon [60] and Vegan [61]. Once calibrated, we select a two hundred years time range for defining our temporal phases. This range is an acceptable time bind regarding the current resolution of radiocarbon dates and associated contexts. To assign each archaeological site or layer into one or more of these chronological phases, we took as a reference the probability mass for each date calibrated at two sigma. We assigned any site/layer to a specific phase if at least 35% of the probability mass fell within. Because this study aims for a higher chronological resolution, after performing several preliminary experiments by constructing time bins with different threshold values, we found 35% to be the maximum value, guaranteeing that almost all the contexts were included in one bin, or two consecutive bins (thus 400 years). Only two contexts were included in three time-bins (therefore 600 years): Atxoste IV, and Castillejos I.

In the first context, the included radiocarbon dates (GrA-13418 and GrA-13419), despite both being associated with Level IV, exhibit a notable chronological separation according to the original publication [62], hence their assignment to three distinct but consecutive temporal windows. A similar situation occurred with artefacts linked to Castillejo’s I. In any case, the radiocarbon dates from these contexts overlapped across consecutive temporal windows, suggesting a plausible inclusion of these contexts within any of them. This rationale guided our decision to retain them in the sample.

Once we have chronologically classified the levels, we include the lithic record associated with the contexts that comprise each temporal bin to construct the archaeological sample. We decided to keep samples from the same site and different cultural levels separate -even when they fall in the same chronological unit, to capture differences in the diachronic history of the site itself. In any case, we think that this assumption allows us to build more high-resolution time bins in comparison with other approaches referring to larger intervals (1500 to 500 years) considering our temporal and contextual range [63, 64]. The next step was to build, according to the previous classification, contingency tables with information on the frequency at which every type appeared in each unit. These tables include types with a frequency greater than 1. As mentioned above, we decided to apply this filter to prioritise those typologies that contained information about possible contacts or interactions that explained their appearance in more than one occasion.

We have employed a simulation method to analyse the impact of sampling variability on archaeological network analyses, as suggested by Peeples and Brughmans [65]. It employs bootstrapping to create random data replicates, keeping sample sizes constant while adjusting geometrics-type probabilities according to the original data’s distribution. This approach facilitates a detailed evaluation of how sampling errors may affect network metrics within geometric similarity networks. By transforming raw data into Jaccard similarity matrices and generating thousands of simulated samples, the script assesses the stability of network measures against sampling fluctuations. The results, visualized through line plots, reveal insights into the reliability of degree centrality rankings and network structure, highlighting the importance of considering sampling variability in archaeological network studies and providing a method to quantify and visualize such variability.

Previous to calculating similarity matrices, the frequency tables were subsequently transformed into binary absence-presence matrices to mitigate potential quantitative variations arising from specific site functions, conservation methods, excavation techniques, and other potential biases. For that reason, despite the Brainerd Robinson-based-frequency coefficient of similarity being the most applied [20], we decided to use Jaccard index [63, 66]. Moreover, when comparing structural characteristics of networks in a diachronic view, previous studies have noted how, in those cases, when the total number of styles recorded (types-based traits in our case) is strongly different over temporal windows, it can introduce biases [66]. Specifically, lower style variability in specific temporal windows could facilitate high similarity values. On the contrary, a broader variability of styles would (a priori) have difficulty drawing high similarity scores. So, because we noted that the number of types of the record changed strongly across temporal samples (see Table 2), to avoid this kind of bias, we normalised the similarity matrices to the most diverse time bin in line with previous applications [66].

**Table 2:** Sample size and number of types present in every time bin.

cal. BP	8600–8401	8400–8201	8200–8001	8000–7801	7800–7601	7600–7401	7400–7201	7200–7001	7000–6801
Nsubstyles	14	14	13	24	21	26	26	22	17
*n* sample	107	226	170	449	417	77	117	79	30

### 2.4 Network construction

Once the adjacency matrices were constructed, the second phase, which covers the network construction, was developed with Gephi [50]. In this case, because this study does not consider the direction of the interaction, we have built an undirected network for every time-bin.

**Fig 3:**
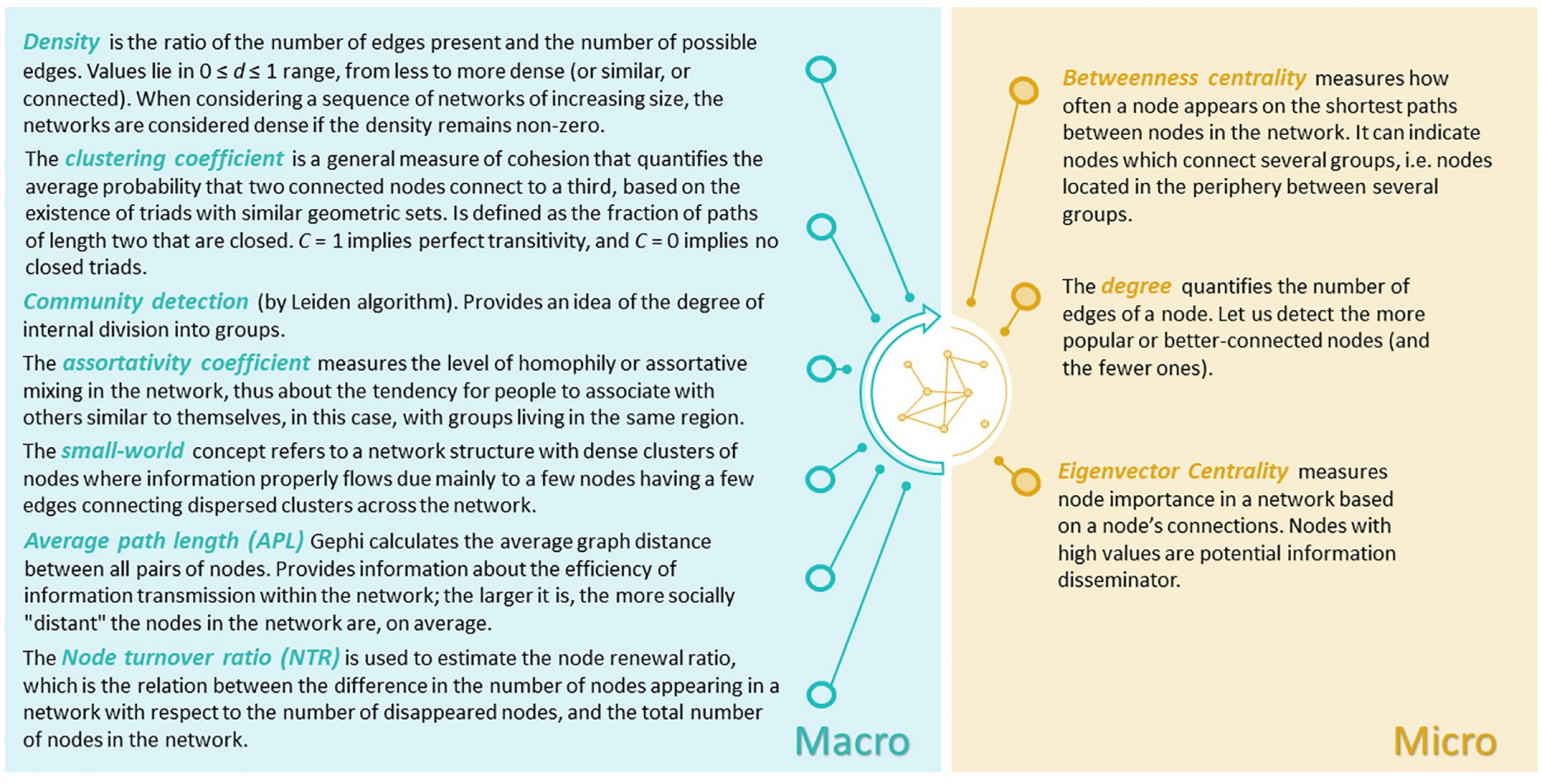
Network metrics calculated according to the scale they report. Several bibliographic references provided information about the definitions, mainly Newman [21], Collar et al., [22], Buchannan et al., [23], and Jiménez-Puerto [67].

As part of analysing the obtained networks, we calculated several structural measures to determine the diachronic evolution. Fig 3 presents the calculated metrics for describing the evolution of the social network. By calculating these metrics, we intend to decode the evolution of the network at different scales, namely, macroscale (structure), which includes those metrics that allow us to know the general structure of the network, and microscale (node level), which consists of those metrics that reveal the role played by the different nodes in the network of which it is a part.

This study uses the triangle method to calculate the clustering coefficient. Despite the basic method, which calculates the coefficient from the average of the index of all nodes in the network (node-level), the triangle method measures the ratio of the global count of triangles and connected triples in the graph [21, 50, 68].

Also, some of the metrics calculated have settings that need to be adjusted according to the data, as is the case of modularity. In this case, we used the Leiden algorithm, a review of the Louvain one, which allows more iterations and corrections in the procedure [69]. Here, we measure the partition quality by the Constant Potts Model (CPM) as a quality function; it is helpful to get the optimal partition of a set of nodes since CPM exclude node mergers that decrease the quality function [69]. After performing several preliminary essays, we found in the following adjusted values the combination that achieves an optimal balance between resolution and the quality of adjustment outcomes for the Leiden algorithm: resolution of 0.02, 1000 iterations, and 100 reboots.

**Fig 4:**
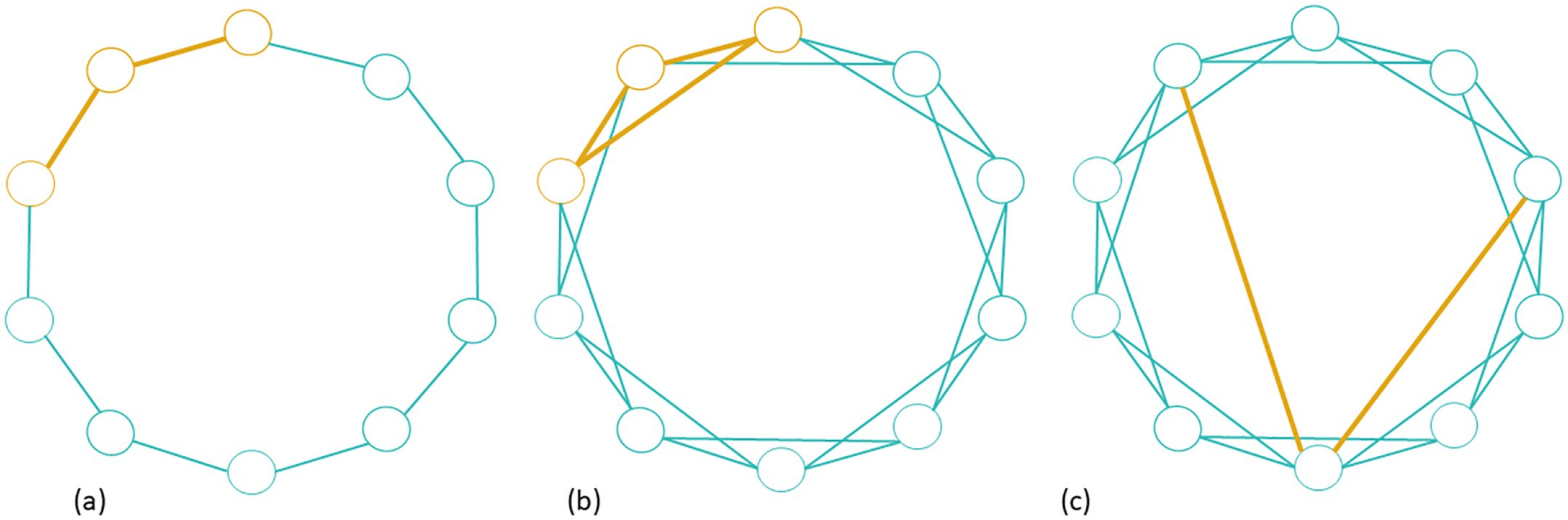
Here are three graphs with the same number of nodes (n = 10). In (a), nodes are connected only with immediate neighbours. In this network, CF = 0; thus, the probability that two connected nodes connect to a third is 0; nodes do not shape communities. Also, APL is high because there are no connections apart from neighbours, so an exchange between non-neighbouring nodes needs to pass through all the edges between intermediate nodes on its path. In (b), additional edges between nodes shaping triads fulfil the clustering coefficient requirement for a small-world network. However, APL is still low. Finally, in (c), the presence of two additional edges shortens the average distance between the nodes that make up the previous network (b), thus fulfilling the missing requirement of "low APL" to be defined as a small-world network.

Concerning the “small world”, the concept defines networks with high clustering coefficients (CF) and low average path lengths (APL) (Fig 4) [21, 23]. In this case, to test whether our networks have a small-world structure, we performed 1000 Erdos-Rényi random networks for every time-bin with the same number of nodes and density as the one observed in the original temporal network [23]. Then, we calculated the clustering coefficient and average path length (APL) values of the random networks and compared them with the original ones based on their distribution representation shaped in boxplots. The intention is to test whether our observed networks have the properties of a small-world network. Thus, following Buchanan’s [23] approach, the clustering coefficient from the original networks should be higher than the random values; however, in the case of APL, the comparison is more complex because even though small-world have a low APL, it is not enough to compare original APL values with random APL values since random ones typically have shorter APL than real networks [23]. Therefore, the original network needs to have a pretty low APL to be lower than the average random APL. Likewise, we could not discard small-world in cases when the original APL were higher than random.

To avoid the previous situation, we follow Buchanan et al., [23], who incorporate in the small-world test protocol some additional calculations, including the coefficient σ from Watts and Strogatz [70]. The small-world coefficient σ is the ratio of the clustering coefficient of the original network and an equivalent random network divided by the ratio of the original APL to the APL of the equivalent random network. Here, we previously calculated such measures for the 1000 iterated random network, so we use the mean for both the clustering coefficient and the APL random of all this created network as the “equivalent” random measure. Except for the small-world test protocol, developed with R studio, we ran the different study steps with Gephi. Code for this and other steps (temporal samples construction) are available in the supplementary.

## 3. Results

### 3.1 Chronological distribution of the data

Table 1 includes the relation between chronological units and the archaeological samples which shape it according to the protocol for constructing time bins. After performing the chronological samples, one factor that stood out was the unevenness of the types in each chronological unit (Table 2). Since the sample size is also variable, we tested the possible correlation between variations in the number of typologies and the sample size. In this case, we used Spearman’s test since the samples did not meet the condition of equality of variance. The result (*p* = 0.763) allows us to rule out the possibility that typological diversity results from differences in sample size.

Following a diachronic description, variability increases significantly from 8000–7801 cal. BP. From then on, variability remained high, with the highest point documented in 7600–7401 and 7400–7201 cal. BP.

**Fig 5:**
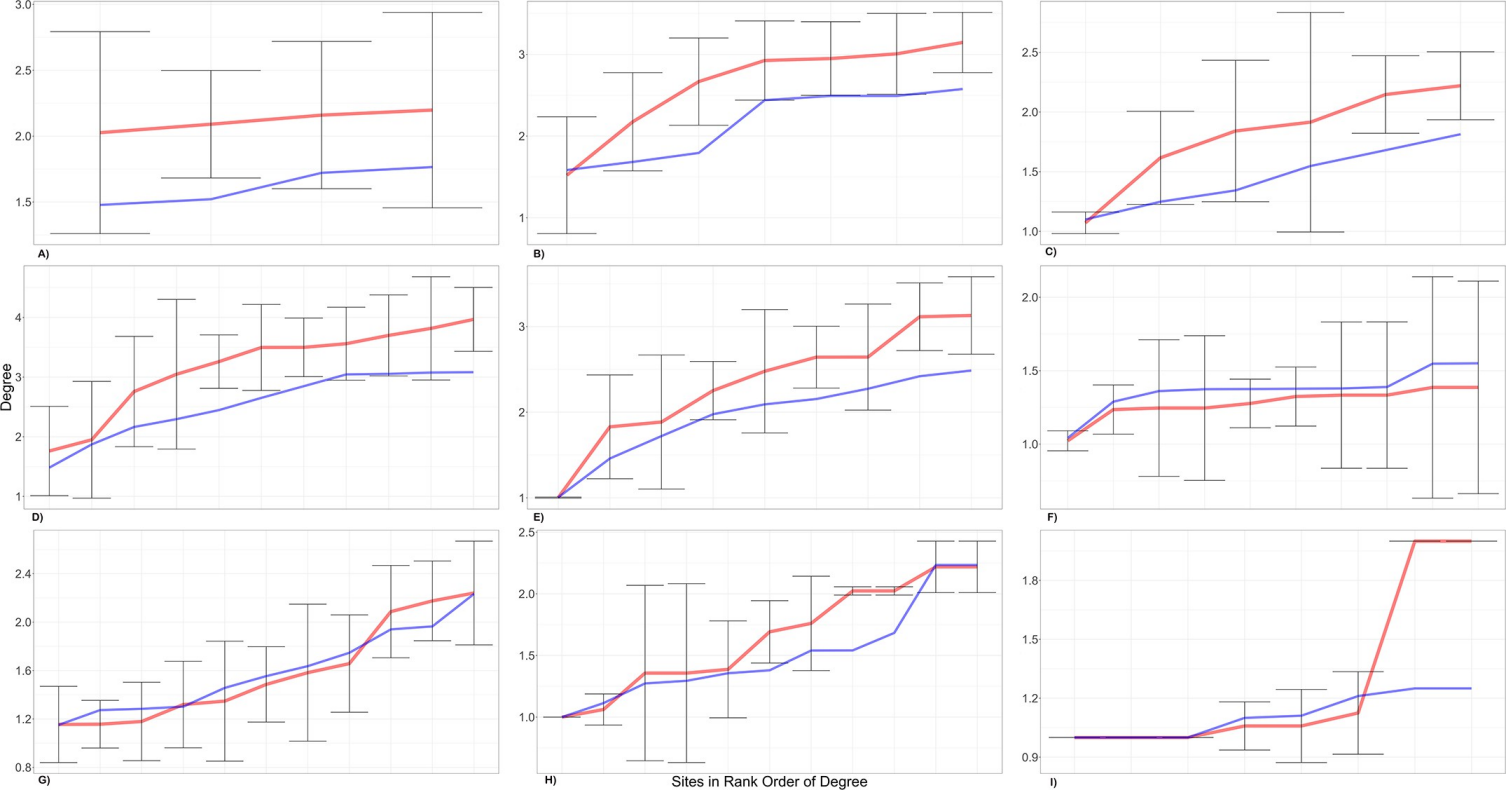
The results of the tests carried out on the representativity of the sample available for network construction.

Regarding the influence of sample size on the network construction, in Fig 5, nine plots, one for each chronological window, have been created. Generally, there is a deal of agreement between the modelled sample and the original. Even within the worst cases (A, B, C and I), just in the I (7000–6800 cal. BP time bin), the number of nodes inside the confidence interval is more than fifty per cent. For all the others, the observed values generally fall within the margins, suggesting that the sample size is large enough to be representative.

### 3.2 Network structural metrics

The results of general structural metrics for the networks are displayed in Table 3. Starting with density; values are high between 8600 and 7601 cal. BP. In density, which measures the ratio of connection and nodes, values higher than 0.5 indicate that more than half of the possible connections are present, given the number of nodes. In this sense, values are lower than that range as of the 7600–7401 cal. BP and, although it increases somewhat in 7400–7201 cal. BP does not recover the previous high values.

**Table 3:** The results of the measurements calculated for the networks.

	*8600*.*8401*	*8400*.*8201*	*8200*.*8001*	*8000*.*7801*	*7800*.*7601*	*7600*.*7401*	*7400*.*7201*	*7200*.*7001*	*7000*.*6801*
***n Nodes***	4	7	6	11	9	10	11	11	8
***n Edges***	6	20	12	53	28	13	29	18	3
***Density***	1	0,952	0,8	0,964	0,778	0,289	0,527	0,327	0,107
***Clustering coefficient***	1	0,95	0,85	0,96	0,92	0,3	0,61	0,56	0
***Gephi APL***	1	1,048	1,2	1,036	1,222	2	1,473	1,6	1,25
***Leiden n clusters***	1	1	1	1	2	3	1	4	5
***Leiden Quality***	0,85	0,85	0,75	0,91	0,9	0,7	0,76	0,82	0,8
***Filter***	NO	NO	NO	NO	NO	NO	NO	NO	NO

*Clustering coefficient* values vary in a similar dynamic. This measure quantifies the average probability that two nodes are connected to a third, thus measuring cohesion. In this case, cohesion decreases over time, with the lowest value present from the 7600–7401 cal. BP. Another measure which informs us about the possible degree of social distance between nodes is the *APL*. Again, values are higher in those networks where the number of communities detected is higher, thus 7600–7401 and 7200–7001 cal. BP.

The previously commented dynamic is consistent with *modularity* or *community detection* development over time. In this case, the number of clusters or communities suggested by the Leiden algorithm is higher in samples from 7600–7401 cal. BP (3 communities), 7200–7001 cal. BP (4 communities) and the latest chronological unit (5 communities). Quality values are generally closer to the optimal (quality = 1) than low values (0).

**Fig 6:**
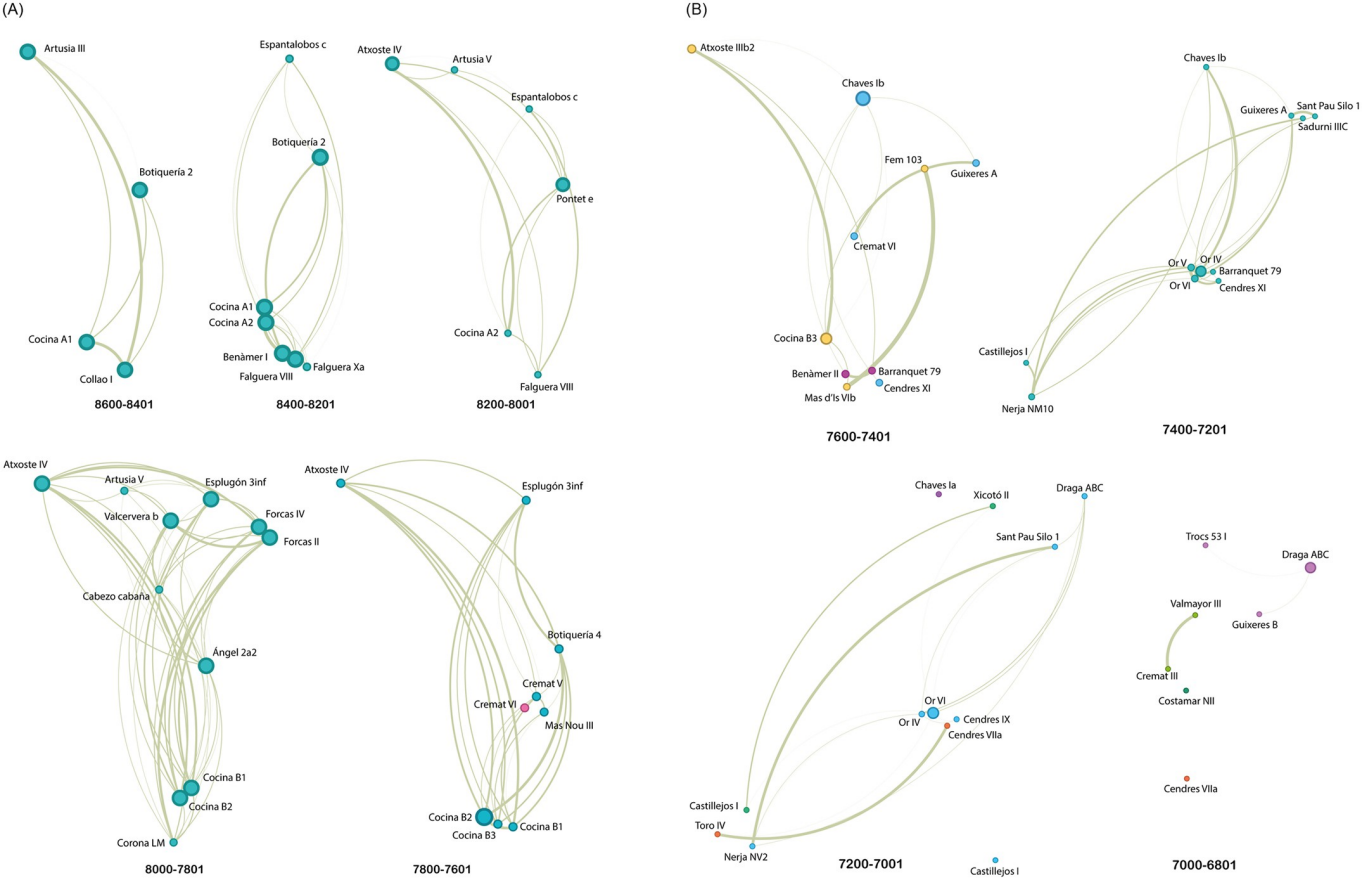
A: Here, networks by chronological unit (8600 to 7601 cal. BP). Nodes represent geometric assemblages, and links represent their similarity. The colour of the nodes indicates membership in a community, and the size indicates betweenness centrality. The colour of the edges indicates the strength of the connection; the more intense the colour is, the higher the relation. B: Here, networks by chronological unit (7600 to 6801 cal. BP). Nodes represent geometric assemblages, and links represent their similarity. The colour of the nodes indicates membership in a community, and the size indicates betweenness centrality. The colour of the edges indicates the strength of the connection; the more intense the colour is, the higher the relation.

Despite the above, according to the microliths data, the first detection of two different communities is 7800–7601 cal. BP (Fig 6A): When we detected differences between Cremat VI and all other levels, including Cremat V. In the other chronological units of Fig 6A, which comprise the entire Mesolithic, the samples are consistent with each other and, therefore, grouped in the same community. As mentioned above, 7600–7401 cal. BP includes a total of 3 communities (Fig 6B), some shaped by both Mesolithic and Neolithic archaeological levels (see the blue and orange nodes in Fig 6B) and others including Neolithic groups (see the pink ones). This richness in the number of communities decreases in the following time unit (7400–7201 cal. BP), where one community groups all the nodes. In 7200–7001 cal. BP the number of communities increases again. In this case, the most populated community is shaped by Draga, Sant Pau, Or (IV and VI), Cendres (IX) and Nerja cave (blue in 7200–7001 cal. BP Fig 6B). Solid edges link the other communities comprising more than one node (oranges and greens). In the last sample, the number of communities is high again, but edges are generally weak; the sample size probably influences both results.

Finally, the renewal ratio of nodes among the calculated time bins is observable by applying the *Node Turnover Ratio* metric (NTR) [66, 71]. In Table 4, we can observe a diachronic variability expressed by, (1) changing signals indicating the growth in the creation of nodes (positive values at: 8400–8201, 8000–7801 to 7800–7601 cal. BP), (2) persistence and continuity (near to 0: 8200–8001 and 7000–6801 cal. BP), and (3) the destruction of nodes (negative values: 7800–7601 and 7000–6801 cal. BP), very noticeable at the latest (7000–6801 cal. BP).

**Table 4:** Results of the NTR calculated for every network.

cal. BP	8600–8401	8400–8201	8200–8001	8000–7801	7800–7601	7600–7401	7400–7201	7200–7001	7000–6801
NTR		0,43	-0,17	0,455	-0,2	0,1	0,1	0	-0,38

### 3.3 Testing the network structure ‘Small-world’

**Fig 7:**
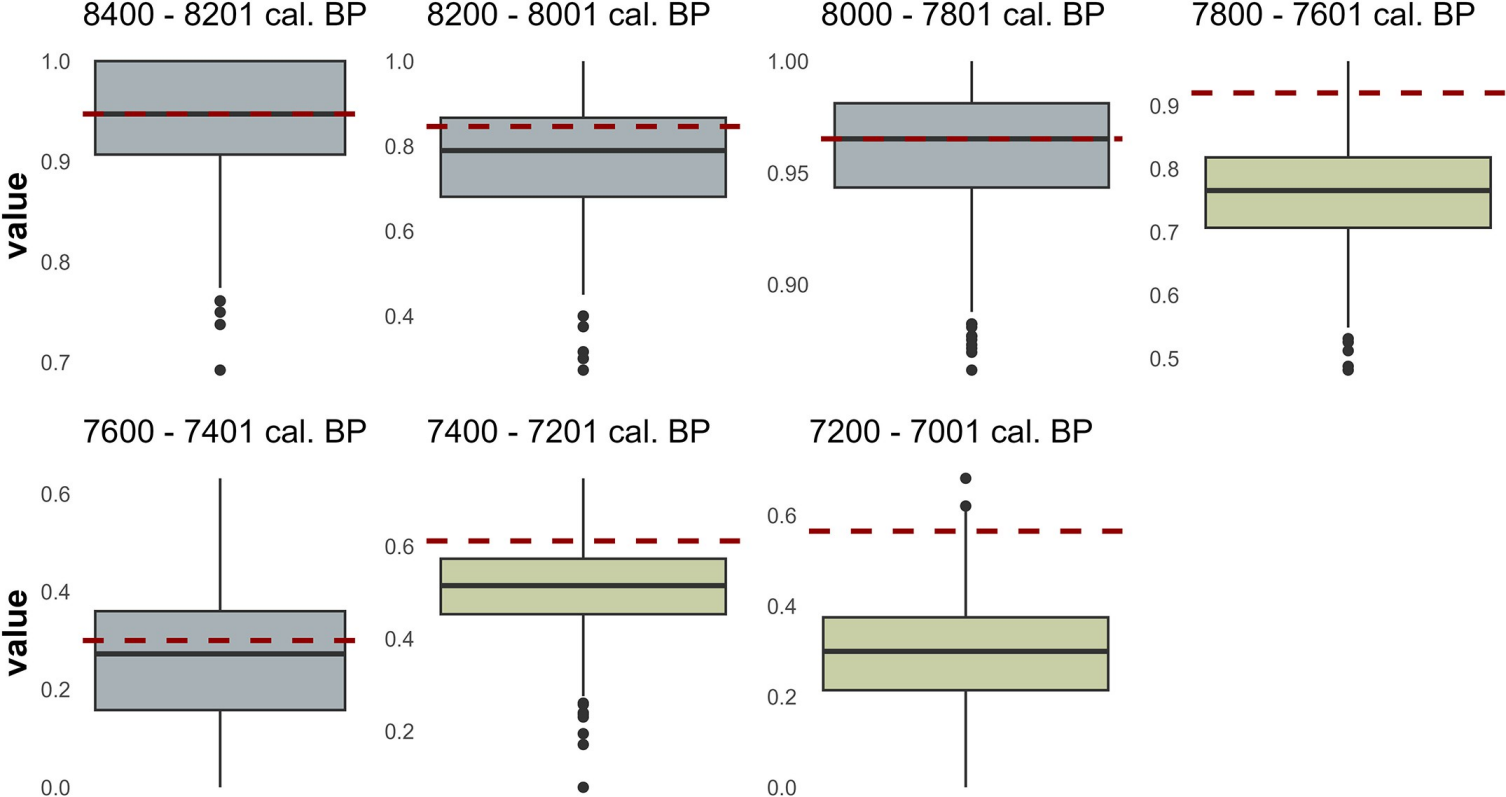
Boxplot for the clustering coefficient values of the 1000 random networks created for every chronological unit. The red dashed line marks the clustering coefficient of the original network.

**Fig 8:**
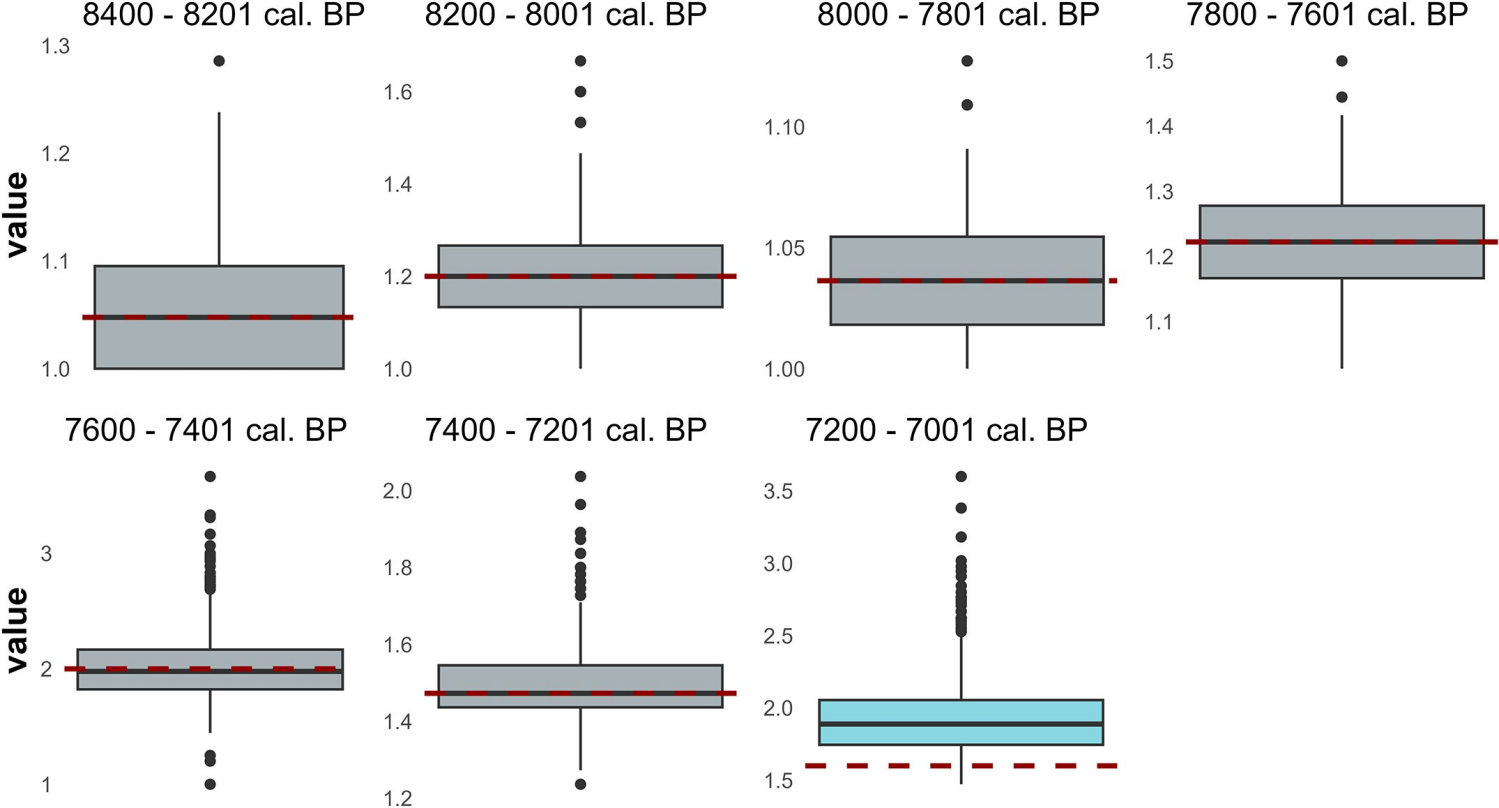
Boxplot for the APL values of the 1000 random networks created for every chronological unit. The red dashed line marks the APL of the original network.

The comparison between the clustering measures of the random networks and the clustering coefficient of the observed microlith networks only detects differences in three cases: 7800–7601, 7400–7201, and 7200–7001 cal. BP. Thus, the structure of those networks does not seem random. In all those cases, the observed networks have a higher clustering coefficient than the random ones, a result that suggests that 7800–7601, 7400–7201, and 7200–7001 cal. BP networks have high clustering coefficient values (Fig 7), which is one of the properties of a small-world network. However, after verifying the APL relation between originals and random networks, we only found differences in 7200–7001 cal. BP networks when the average path length is lower than in random networks for that chronological unit (Fig 8). Regarding the coefficient σ, considering that the author indicates in the original text that measures greater than one is equivalent to a network with small-world properties [70], the network which better responds to that definition is 7200–7001 cal. BP (Table 5), which is consistent with the previous one.

**Table 5:** Results of the small world coefficient σ [70] calculated for the original networks.

	*8600*.*8401*	*8400*.*8201*	*8200*.*8001*	*8000*.*7801*	*7800*.*7601*	*7600*.*7401*	*7400*.*7201*	*7200*.*7001*	*7000*.*6801*
Watts and Strogatz’s σ [70]	1	1,002	1,1	1,002	1,209	NaN	1,223	2,35	NaN

From an overall assessment of the results obtained in (1) the comparison of the clustering coefficient, (2) the APL between the original and the random networks and (3) the values of the small-world coefficient, we confirm that the network generated in 7200–7001 cal. BP presents the properties of a small-world structure; we do not rule out, moreover, that the networks generated in 7800–7601 and 7400–7201 cal. BP present, in addition, these properties. While it is true that the latter two share APL with the random nets, we consider it prudent not to overlook the fact that, in the first place, the APL they present is low despite not being lower than those of the random nets. It should be recalled here that random networks have low APL values per se. Moreover, their σ values are also greater than one, although they are closer to unity than in the case of 7200–7001 cal. BP.

### 3.4 Micro-scale metrics

Fig 5A and 5B supply information on the *betweenness centrality* of the nodes at each chronology. Although it is a measure that allows us to evaluate dynamics at the micro-scale, we first comment here on a general evolution of the equilibrium or imbalance in which the nodes find themselves concerning this measure over time, and then point out, when this is the case, those nodes that seem to have a prominent role in the flow of information. Thus, the first moment when the betweenness centrality values have a certain unevenness is in 8200–8000 cal. BP, with Atxoste level IV and Pontet level e as the sites with the highest values. This is followed by 7800–7600 cal. BP, where phase B of the Cocina site hosts the highest values of the total sample. After that, the values remain unequal between nodes. In the following window, 7600–7400 cal. BP, it is the Chaves Cave (phase Ib) and, secondly, the B3 phase at Cocina that show the highest values. Between two and four hundred years later (7400–7200 cal. BP), the site of Cova de l’Or, located in a central Mediterranean position, contains the highest measurement values. This situation is similar during the years 7200–7000 cal. BP.

The *eigenvector centrality*, on the other hand, shows markedly unequal values from 7600–7400 cal. BP, when sites Chaves Ib, Cocina B3 and Atxoste IIIb2 hold the highest values (1, 0.76 and 0,67, respectively). Before this, the values remained relatively similar to each other (see S1 Table). In 7400–7200 cal. BP, Cova de l’Or maintains high values in its different phases with EC values between 0.9 and 1. Sites of Chaves (Ib), Nerja and Guixeres (A) follow the previous one, with EC values between 0.77 and 0.65. Incorporating the latest as nodes with high centrality values is a novelty concerning the BC values. These sites, along with La Draga and Sant Pau, host the higher values during 7200–7000 cal. BP (S1 Table).

## 4. Discussion

Exploring patterns of cultural variability in space and time constitutes a challenge for archaeological research considering the nature of data (scarcity and inequality of the samples) and the degree of palimpsest that currently forms contextual information. Our attempt here focuses on social connectivity using SNA in a *longue durée* view trying to evaluate continuities and changes in cultural links among hunter-gatherer societies and those emerging from the dispersal of agricultural groups. To do this, we focus on the Mediterranean Iberia, which constitutes an appropriate data laboratory thanks to the great number of published assemblages covering the neolithisation process understood in a wide sense, from the middle of the IX millennium to the beginning of the VII millennium cal. BP [72–75]. The selection of geometric projectiles has provided a suitable proxy for comparing the entire temporal range considered. For the first time at this regional and temporal scale, we point to these objects to explore culture variation from SNA, as has also recently been applied to pottery decoration patterns, and/or ornament assemblages [63, 64, 66].

We have prioritized sampling accuracy according to contextual and chronological criteria to obtain consistent information that mitigates potential biases introduced in a large amount of data (without validation criteria). Nevertheless, we assume other biases as the reduced number of potential actors (nodes) (see sample test results). We think that their wide spatial distribution maybe could diminish some effects as visible in the graphs obtained from the general cultural connectivity observed. In addition, potential confounding factors such as the effect of the research intensity and/or the assumed spatial boundaries are considered in the discussion.

### 4.1 Structural and level network metrics

Focusing on structural patterns, we observe various changes that reveal apparent stability among the temporal windows relating to the last Mesolithic general phase and disruptive shifts triggered by the Neolithic arrival. Concerning the Mesolithic, the high values offered by the density and clustering coefficient metrics (near 1) (from 8600 to 7801 cal. BP) reflect the high connection observed from the variability of geometric projectiles. APL results also indicate similar path lengths reinforcing some kind of persistence signal from cultural patterns. Accordingly, we can hypothesise how information connectivity could be explained by subsistence patterns and social actions that promote behavioural strategies invoking some kind of homophily [20] expressed by low structural variability (traduced in a high diachronic homogeneity conforming one unique community). Apparent homogeneity must be understood involving local (minimal bands) and regional groups (maximal bands) where information and social actions can boost long-distance knowledge dispersal [76]. Other networks related to some precious objects such as Mediterranean ornaments (*columbella*) reveal the long distances paths covered at an interregional scale [63]. This Network mobility can invoke kinship relationships and other social actions (ceremonials), but also information, expressed by mobility patterns directed to minimising risks promoting contacts among “maximal bands” [76].

Regarding the node-level analysis, the distinguishing weight among nodes is visible only in 8200–8000 cal. BP (Atxoste IV and Pontet b), although the next interval redistributes the weight more regularly (8000–7801 cal. BP). The 8.2 event has been recurrently discussed as responsible for some of the perceived changing patterns at the microregional level [77, 78], although the possibility that its effects are not conclusive has also been pointed out [79–81]. In the next time bin (7800–7601 cal. BP) we find a significant weight, both betweenness and eigenvector centrality, highlighting the possible special role of Cueva de la Cocina in the central Mediterranean area [35, 43].

From 7800–7601 cal. BP, some slight shifts emerge, coincident with the latest well-known hunter-gatherer settlement remains, and probably with the echoes of the first signals of the Neolithic dispersal from the Iberian Mediterranean shores [5, 9, 10, 26, 44, 82]. Density metrics show now a slight decrease (0.778) that will be dramatically followed by a clear drop coincident with the 7600–7401 cal. BP period.

From the spread of the Neolithic, metrics over structural networks indicate a breakup moment (circa 7600 cal. BP). This temporal window displays a reversal in density and clustering coefficient metrics, which show low values, and the detection of 3 communities among nodes. The observed reversal metrics conform to the new spatial pattern regarding node distribution fostered by the Neolithic diffusion (in a first step mainly around the Mediterranean corridor), and, as expected, with a new driver for cultural diversity. Interestingly, the metric has captured the connectivity between the latest Mesolithic nodes (Cocina B3 and Atxoste IIIb2), visualizing the coexistence of diverse communities that fit well with differences among hunter-gatherers and first farmers. Nevertheless, modularity reveals three potential associations that merge with some Neolithic sites, even if derived from low connectivity. It also seems outstanding the group conformed by Barranquet UE79 and Benàmer II. Barranquet II constitutes the first identified pioneer impressa site in the Mediterranean Iberia [83]. This aspect is relevant according to current data, although other identified episodes (as Mas d’Is Ib) wouldn’t fit with the same group. Other Early Neolithic sites, in this case, Guixeres A, Chaves Ib, and Cendres XI make up a group including early cardial pottery tradition. Consequently, the interval 7600–7400 cal. BP depicts in some manner the dissolution of the Mesolithic links and the punctuated fast (and arrhythmic) advance of the Neolithic communities. The lack of sample data for Andalusia is likely the effect of the assumed filter criteria given that the neolithic dispersal signals are well documented in this temporal span [37, 84, 85].

In any way, there seems to be a good fit with the widely accepted neolithisation model; that is the expansion of Neolithic socioecological dynamics through the Mediterranean coast from the Northeast (current data fit well with this hypothesis but see also possible open scenarios from the South [6]) and extending to the inner (mainly through fluvial corridors: Ebro valley) and coastal areas involving cultural interactions with hunter-gatherer population [7, 12, 74, 76, 82, 86].

The consolidation of the Cardial Neolithic world has been captured in the next interval (7400–7201 cal. BP) when only one community is detected, coinciding with higher values from density (0.527) and cluster coefficient (0.61), so inferior to previous time-bins. The image of the depicted nodes shows the spread around the Mediterranean corridor including Andalusian sites at the provinces of Granada (Castillejos I) and Malaga (Nerja NM10). A more homogeneous pattern can be assumed, where Cap de la Nau/Serpis valley area (North Alicante and South Valencia provinces) retains higher betweenness weight, particularly expressed at Cova de l’Or site. This information is consistent with the consolidated pattern of Early Neolithic settlements in this central Mediterranean area, exhibiting a first expansion from the pioneering arrival, and further expanding widely during the second half of the VIII millennium cal. BP [44].

The next time bin (7200–7001 cal. BP) starts to anticipate the disintegration of Cardial culture wear, also attested in the regionalisation of decorative styles from pottery records [66]. The structural metrics point to the fall in density although the clustering coefficient maintains similar previous time-bin results. The nodes involved conform to four different communities according to cultural variability derived from geometric tools. The spatial pattern reveals coastal and inner differences expressed by most of the sets of nodes covering the Mediterranean corridor, when also central Mediterranean shores (particularly Or VI) conform the highest betweenness, facing some inner residual relations (Castillejos I and Xicotó II), and the isolation position of Chaves Ia in the Ebro valley. The more recent temporal window evaluated (7000–6801 cal. BP) shows the final breaking up of the connectivity, that could be linked with the consolidation of regionalisation in cultural terms (in any case, from a minor number of nodes).

### 4.2 Network dynamics, demographic trends and small world phenomena

Focusing on a dynamic view, the information is particularly relevant thinking about some general structural aspects concerning demographic and socioecological trends revealed from regional Mesolithic and Neolithic literature [79, 82, 87, 88].

As previously commented, the NTR already pointed to a diachronic variability with different stages, including node growth (8400–8201, 8000–7801 to 7800–7601 cal. BP), continuity periods (8200–8001 and 7000–6801 cal. BP), and stages of node destruction (7800–7601 and 7000–6801 cal. BP).

In this line, changes in structural social networks must be related to demography as a main factor for triggering cultural evolution, and consequently, to the connectivity expressed from social relation links. Focusing on Summed Probability Distribution of calibrated dates (SPDs), at the Iberian scale [79, 82, 89–92], but also when we point to regional approaches [87, 90, 93], there is a visible increasing pattern starting with the last Mesolithic and, particularly, the boom effect propelled by the Neolithic expansion. Fluctuations detected include significant bust signals at the end of the Early Neolithic, as has also been described in other European regions [94].

We are interested in arguing some relevant aspects resulting from comparing some network structural metrics and trends in demographic patterns. Because of the low number of nodes involved in our analysis (compared with other network works focused on different selection criteria), exploring changes in network trending size does not seem relevant. Instead, our interest is to evaluate how demographic proxies and network metrics correlate. Firstly, we point at the turnover produced during the interaction event among hunter-gatherers, and first farmers and herders (circa 7600 cal. BP). The increasing demographic signal coincides with the restructuring produced at the network structural metric level (lowest density and cluster coefficient, increase of communities and largest APL) and the subsequent stabilization at regional scales (7600–7201 cal. BP). If some “founder effect” and interbreeding pattern are plausible explanatory hypotheses at first (7600–7401 cal. BP), the subsequent regularisation would claim for possible homogeneity process (one community recognised) regarding Cardial Neolithic networks consolidation (7400–7201 cal. BP). Secondly, the fall in demographic curves from SPDs and the end of the VIII millennium cal. BP would be related to the disintegration of Early Neolithic networks and the increasing regionalisation. This might have been promoted by internal factors produced by cumulative socioecological drivers originating from local dynamics and their multiscalar effects predicted by Complexity Adaptative Systems [79]. These last signals coincide with the analysis of social network structure obtained from pottery decorative techniques in the Iberian Early Neolithic record as the lowest network size and negative values of NTR start around 7000 cal. BP [66]. Claims for external factors such as climatic events (7.1 event) [79] do not seem to be the main driver in the current state of data.

At this point, some comments on the emergence of a small-world network structure in at least one of the temporal samples are due. Our results show that a small-world structure could explain similarities in at least 7200–7001 cal. BP. The definition of the small-world network includes having a high clustering coefficient and short path lengths, and, despite the sample having a CC lower than the ones shown in other periods, it is higher than the one shown by the thousand equivalent random networks, as well as a lower APL, not to mention its high σ coefficient. The properties of small worlds support the flow of information in a clustered network, so it is appealing to think that this structure is present and supports some level of information flow in this phase in which regionalisation starts. Regarding additional micro-scale measures, we found higher values in eigenvector centrality in Draga, Sant Pau, Or, Cendres and Nerja. Due to the coastal position of those sites, which shapes the primary connection between distant areas as well (Fig 6B), we wonder whether the Mediterranean continues to play an essential role in path connectivity, thus helping to keep geographically dispersed populations linked. This hypothesis associates the coastal transport factor with groups who, generations before, colonised the territory mainly similarly, thus with populations that were probably good navigators. We consider the previous hypothesis for future contrast with a larger sample, especially concerning the inner territories.

Along these lines, it could be helpful to analyse what about small-world according to other archaeological materials, such as ceramic remains, in which network analysis [66] has observed similar tendencies in general network measures; e.g. the disappearance of cardial styles in interior areas may be a cause of isolation concerning coastal areas from circa 7400–7300 cal. BP, a situation followed by measures that suggest a scenario with more dynamic areas in the interior of the Iberian peninsula and certain stagnation on the coast (7300–7100 cal. BP), and finally a collapse (7100–6800 cal. BP) in which the density of the network decreases, and a progressive division of the network into clusters takes place. In our case, the possible presence of a small-world network structure does not imply a greater flow of information than the other phases. Further, small-world does not imply that this phase presents a higher cultural homogeneity than in previous dates. We propose here that, when regionalisation appears to be increasing, a small-world structure where nodes on the coast present the most robust links could explain the similarities documented in the record. The network analysis in general and the detection of small-world structures, in particular, are often related to the raw material exchange [23]. Thus, what could it imply that cultural networks (cultural information in this context) were small-world? The answer depends on how one understands the presence of this similarity in the geometric toolkit. Here, we will understand similarities as homogeneities promoted by maintaining contacts between culturally related groups. One must imagine a complex social network that promotes intra-regional contacts but coexists with some inter-regional connections (maybe facilitated by coastal movements).

## 5. Conclusion

In this paper, we have evaluated social structures from an SNA approach accounting for variability in geometric projectile tools in the framework of the neolithisation process in the Western Mediterranean. We point at a long-durée view for plotting diachronic variability expressed by shifts in structural and node-level metrics reporting about information flow derived by our *ad hoc* types. We are based on the premise that resulting patterns of cultural variability can provide powerful data for understanding cultural change and variation when horizontal transmission processes, among others, constitute a crucial factor. Our approach relies on the accurate selection of well-dated contexts from the designed criteria of validation, bearing in mind that the reduction of spurious sample bias effects, and the more refined chronological time-bins, will contribute to more refined results. In addition, it constitutes a proposal that introduces, for the first time, geometric projectiles as a good proxy for exploring variability considering SNA in the context of the neolithisation process from a Mediterranean perspective. Other approaches pointing to these issues have used pottery and/or ornaments, producing interesting questions when comparing results [63, 64, 66]. Although unequal geographic and temporal scales hinder direct comparison, it is possible to stress some relevant aspects.

First of all, when we compare the aforementioned SNA works accounting for ornaments, pottery decoration techniques, and in our case, geometric projectile types, results have referred to different evolutionary stories. For ornaments, both the Mesolithic [63] and Neolithic [64] revealed more spatially homogeneous patterns and temporal persistence. In contrast, pottery decorative techniques show a more heterogeneous variability through time, conforming to some kind of regional identity patterns [64] that fit well with several explaining evolutionary hypotheses about Neolithic spread and consolidation. Accounting for pottery records, and considering a similar geographic and temporal scope (The Central and Western Mediterranean), Pardo-Gordó and colleagues [95] have obtained slightly different results based on cultural distance matrices and cladistics. This study reveals a weak signal of isolation-by-distance and a clearer pattern of phylogenetics structure. Nevertheless, as derived by different databases, context selection and temporal windows, differences among studies persist, highlighting the importance of improving approaches and promoting discussion about them.

Secondly, we want to stand out how lithic projectiles constitute a good proxy for investigating patterns of cultural change, proving that different approaches produce complementary results to tackle evolutionary trajectories [23, 25, 96–98]. We point here to a specific type of projectile (geometric projectile) to contribute to these issues, revealing interesting results as possible signals of the emergence of “small world” phenomena among others. The increase in sample records constitutes a current goal to contribute to a better understanding of patterning results.

Finally, we emphasise the interest in comparing different archaeological material proxies to address population structure in cultural data. These approaches can contribute to understanding cultural data as a consistent collection of archaeological units sharing evolutionary stories, or ensembles shaped by independent units (as would be the case if we confront our general compared results). To do this, it is necessary to promote quality in published data (both in detailed publications of well-dated and defined archaeological contexts and material characteristics) and expand the number of works thinking about evolutionary questions.

## Supporting information

S1 File Sites’ sampled details. (DOCX)

S1 Table Eigenvector centrality values. To ensure full replicability of the study, all data necessary to replicate the analysis and the script will be available to the public under a Creative Commons license at the GitHub repository at https://github.com/MBarreraCruz/SNA_Geometrics.git.
(XLSX)
