# Supplementary material for: Weaving social networks from cultural similarities on the neolithisation process in the Western Mediterranean: Evolutionary trajectories using projectile tools
Source: PLoS One. 2024 Jul 30;19(7):e0306027. doi: 10.1371/journal.pone.0306027 (PMC11288410; doi:10.1371/journal.pone.0306027)
Supplement: S1 File — (DOCX) [file pone.0306027.s001.docx]

# Sites

This section includes information about archaeological levels, geometrics, and dates incorporated in the sample analyzed. Contexts were assigned to one of the Integrity Levels according to (A) the context integrity and (B) the C14 dates available for each unit (see details in Figure 1). As a result, several contexts respond to Level 4 of integrity, and some refer to a higher resolution (Levels 2 and 3). For every context, one can find the integrity level as ‘L-‘, followed by the number. We include here the source where information about the geometrics is available unless this work’s authors collected data. This study does not include archaeological contexts identified as Level 5. Details about the integrity levels of the archaeological data are available in the file ‘Geo_Redes_PUBLISH.csv’ provided together with the script.

Figure 1: Criteria followed to incorporate or discard archaeological levels from the sample.

The number of geometric refers to the total number included in the first step of the study, thus previously to the filters applied. On that point, we limit the discussions concerning the consequences of the appearance filter to those levels excluded from the sample due to its application.

Artusia (Unzué, Navarra)

The archaeological site, discovered in 1996, is located in a rock shelter between the Alaiz mountains and the Unzué cliff, in the pre-Pyrenean basin of the Ebro River. Archaeological work on the site took the form of two consecutive campaigns held in 2009 and 2010, under Dr. Manuel Rojo Guerra (University of Valladolid). Its stratigraphic package comprises five Mesolithic levels or phases, dated between 7461-7171 and 6205-6009 cal BC [1]. This study incorporates archaeological material from Phases **III** (L-4) (4 geometric artifacts) and **V** (L-4) (2 geometric artifacts) [1], with Phase IV excluded due to the absence of datings and diagnostic material. The material culture of Phase III has been associated with a dating of 7680 ± 40 (Beta-374431), resulting in the temporal time-bin of 8600-8401 cal BP. In contrast, Phase V is linked to a dating of 7210 ± 30 BP (Beta-379670), placing it within the temporal time-bin of 8200-8001 cal BP.

Abric de la Falguera (Alcoi, Alicante)

The archaeological site underwent its initial excavation in the early 1980s of the 20th century, followed by subsequent excavations conducted from 1998-2001 [2]. Recent investigations have solidified the foundational elements of the archaeological sequence. Within this framework, a comprehensive delineation has emerged, encompassing eight cultural phases, with a Mesolithic phase forming the foundational stratum and a contemporaneous stage situated at the pinnacle. This study includes lithic samples from that Mesolithic phase, specifically from UEs 3188 and 3200 in levels **VIII** (L-2) (2 microliths) and UEs 3151 and 3152 in level **Xa** (L-2) (2 other microliths) [2]. While the first one is represented in 8400-8201 and 8200-8001 cal BP samples from the C14 date of 7380 ± 40 BP (Beta-267441), the second one is only included in 8400-8201 cal BP with a C14 date of 7526 ± 44 BP (AA-59519).

Abric del Xicotó (Alòs de Balaguer, Lleida)

Archaeological investigations of the shelter commenced in 1999 under the direction of Dr. María Mercè Bergadà and Xavier Mangado. Subsequently, in 2013, Xavier Mangado and Marta Sánchez de la Torre resumed the excavations, leading to five additional campaigns. The stratigraphy reveals a superficial layer blending prehistoric and modern items as well as the distinct Levels I and II. Level I, chronologically situated in the Bronze Age based on dates, lacks diagnostic elements beyond ceramics with rough surfaces and finger-impressed cordons. In contrast, Level II exhibits dating and characteristics indicative of the Early Neolithic, featuring three post-holes and three combustion structures. Despite the authors' acknowledgment of the sedimentological challenges in distinguishing between the two levels [3], this study incorporates four geometric microliths found in level **II** (L-4) [3], as they align with the dating and materials identified in that stratum. Chronological dating for this level is derived from a sample taken from one of the hearths (Beta-382458), which, with a dating of 6210 ± 40 BP, places the assemblage within the timeframe of 7200-7001 cal BP.

Ángel 2 (Castellote, Teruel)

The Ángel 2 shelter, subject to various interventions by different teams between the years 1986-1992 and 2000-2009, includes within its stratigraphy layers associated with the cultural phases of Mesolithic Macrolithic (level 2b), Mesolithic Geometric (level 2a), and Neolithic (level 2a1). Additionally, the shelter exhibits rock art on its walls [4]. This study incorporates 6 geometric microliths from the Ángel **2a2** level (L-4) [5], chronologically situated within the time frame of 8000-7801 cal BP, as determined by the radiocarbon dating of 7120 ± 50 BP (Beta-286820). The recovered materials align with the assigned cultural affiliation, although certain radiocarbon dates suggest potential stratigraphic inversion, possibly resulting from the excavation of hut floors or other structures by Neolithic groups that utilized the shelter [4].

Atxoste (Álava, País Vasco)

The rock shelter of Atxoste is situated in the upper Ebro Valley. Archaeological works on the site were carried out between 1995 and 2006 under the direction of Alfonso Alday [6]. Radiocarbon dates indicate occupation dynamics from the final Magdalenian to the Neolithic phase, including some continuities during the Mesolithic [7]. Here, we incorporated microlithic data [6] from Mesolithic units **IV** (62 pieces) and **IIIb2** (22 pieces) (L-2). In the first context, the included radiocarbon dates (GrA-13418 and GrA-13419), despite both being associated with Level IV, exhibit a notable chronological separation according to the original publication [7], hence their assignment to three distinct but consecutive temporal time bins (8200-8001, 8000-7801 and 7800-7601 cal BP). In this case, we filter dates according to recent information from dates issues published in Soto *et al*. [8]. With a C14 date of 6710 ± 50 BP (GrA-13417), the pieces from unit IIIb2 are included in 7600-7401 cal BP.

Barranquet (Oliva, Valencia)

Situated in Oliva (València), Barranquet stands as an open-air archaeological site formed within paleochannels during the Ancient Neolithic period, approximately 3 km from the coastline [9]. The impetus for archaeological exploration at this site originated from an urban development project, revealing four distinct occupational levels, including contemporaneous structures. The Neolithic stratum occupied an area of 305 m2, where, notably, anthropic structures were not discerned, likely attributed to post-depositional processes. Significantly, the Barranquet site has documented *impressa* decoration pottery, accompanied by a radiocarbon dating of 6510 ± 50 BP (Beta-221431) based on *ovis aries*. Additionally, 2 geometric microliths from the Ancient Neolithic level (**UE79**) (L-1) have been identified at this location. Due to the C14 date, this sample has been incorporated into 7600-7401 cal BP cultural data.

Benàmer (Muro d’Alcoi, Alicante)

The commencement of archaeological interventions in this open-air site in 2008 and 2009 was prompted by the construction of a motorway [10]. The stratigraphic sequence in a wide area reveals distinct and intermittent occupational phases spanning from the IX millennium cal BP to the fourth-third centuries BC. This timeline encompasses Mesolithic and Neolithic levels, with a notable hiatus of approximately 900 radiocarbon (14C) years. Particularly, this study includes 33 microliths from **level I** (except the erosive UE 2213) (L-4) and 3 pieces from **level II** (L-4) [10]. While the first one is linked to Mesolithic human groups, and includes in the temporal bin 8400-8201 cal BP due to the date of 7480 ± 40 BP (Beta-287331), in level II is found the Ancient Neolithic. This level presents combustion structures and consumption and production areas with a 14C of 6575 ± 50 (CNA539) and it was included in the 7600-7401 cal BP sample.

Botiquería dels Moros (Mazaleón, Teruel)

The excavation of this shelter occurred through several campaigns in the second half of the 20th century, initially under the direction of J. Tomás Maigi. Subsequently, E. Vallespí supervised the project, and decades later, Ignacio Barandiarán [11], from the Department of History at the University of Zaragoza, took charge. The levels discussed here, Botiquería 2 and 4, were documented during the latest conducted campaign. Botiquería **2** (L-4) is characterized as a dense level exhibiting multiple signs of hearth activity and a high frequency of archaeological material. Dated at 7600 ± 50 BP (GrA-13265), the associated sample of 43 geometric artifacts [11] from this level falls within the chronological time-bins of 8600-8401 and 8400-8201 cal BP. Level **4** (L-4) represents a stratum with less density than the preceding one, yet it also displays evidence of fire activity. Dated at 6830 ± 50 BP (GrA-13267), the associated sample for this level (16 pieces) [11] is situated within the timeframe of 7800-7601 cal BP.

Cabezo de la Cruz (La Muela, Zaragoza)

Fieldwork at this open-air archaeological site commenced in 2004, leading to the identification and documentation of a Mesolithic camp. Based on the gathered data, the occupation and abandonment sequence proposed by researchers [12] encompasses the construction of a hut dated to the late 8th millennium BP, with subsequent episodes of occupation until an estimated abandonment around the beginning of the 7th millennium BP. In this study, a total of 8 microliths [12] documented in different units of the **hut** are selected, including an occupation level (UE 1441) and two upper levels (UE 1351 and 1398, the latter being a subunit of the former identified post-excavation) described as combined levels displaced by occupation and collapse of the hut (L-3). For the chronological localization of the artifacts within the temporal time-bins used, a dating of 7150 ± 70 BP (GrN-29135 in UE 1397) has been incorporated. This date, derived from remains of one of the supporting posts of the structure, despite dating the moment of hut construction or rather the fall of the post itself, is placed in an upper unit due to its final position after the collapse. Thus, the sample is included in the timeframe of 8000-7801 cal. BP.

Casa Corona (Villena, Alicante)

Archaeological material from this open-air site was excavated in 2008 by the enterprise Arpa Patrimonio S.L. The archaeological sequence can be summarized as a combination of both natural and anthropogenic units. In the initial occupational phase, attributed to the Late Mesolithic epoch, a series of archaeological deposits emerged within a grey-brown dark sandy matrix containing ashes and organic materials. This phase is characterized by the presence of 16 distinct features, comprising 14 hearths and 2 pit graves housing individual inhumations. Subsequently, the site witnessed a reoccupation during the Chalcolithic period, marked by the establishment of a settlement characterized by pit structures [13]. We include a total of 12 geometric microliths [13] from the **Late Mesolithic** level in the sample (L-4). Due to the available dates for both burials associated with the phase, 7070 ± 40 BP (Beta-272856) and 7116 ± 32 BP (OxA-V-2392-92), these artifacts are part of the assemblage assigned to the chronological time-bin of 8000-7801 cal BP.

Caserna de Sant Pau (Barcelona)

The archaeological works conducted in the late 1980s, 1990-1, facilitated the recovery of a collection of structures and materials dating back to the mid-6th millennium BCE, with continuity extending until the first millennium BCE [14,15]. The sequence includes seven distinct levels, and it is the base level IV which housing remains the oldest occupation of the site. Sant Pau del Camp **IV base** is shaped for several pits whose dates and cultural material could indicate different occupations. Available dates came from silos 1, 2, 9 and 10. Of them all, silos 9 and 10 present the earliest dates as well as some *impressa* ceramic fragments, pointing to a possible more archaic occupation [16]. Here, to join the geometric included in the sample (**silo 1**) (L-2), we used a date of 6290 ± 50 BP (Beta-236174) located in the context. Thus, the geometric is located in the time bins 7400-7201 and 7200-7001 cal BP.

Castillejos (La Peña de los Gitanos, Granada).

In this open-air-site, the stratigraphic sequence was comprehensively outlined through interventions conducted in 1971 and 1974, with subsequent campaigns in 1991 and 1994 contributing additional information [17–19]. The established sequence suggests an intermittent human habitation spanning from the 8th millennium cal BP [20]. For this article, a total of two geometric microliths have been selected. As a chronological reference, we incorporate the total of the short-lived samples dated for **phase I** (L-4), thus 6310 ± 45 (Ua-36215), 6120 ± 40 (Ua-36213), 6260 ± 45 (Ua-36214), and 6140 ± 45 (Ua-37844). Because details about the context of the pieces are not available, we include both geometrics in every time bin where the C14 dates were included, thus 7400-7201, 7200-7001, and 7000-6801 cal BP.

San Sadurní (Begues, Barcelona)

The site, a 200 m2 long cave, is located near a wide terrace in the middle of the Massís del Garraf [21]. Archaeological works on this site conducted since 1980s and continued from 1992 [22]. Human occupations in the cave are documented in a wide stratigraphy ranging, including levels from postcardial to medieval chronologies. This study includes one geometric from the **layer 18** (L-1) [21], a cardial sepulchral level dated on C14 6340 ± 35 BP (CNA-4693.1.1), 6370 ± 30 BP (Beta-398966) and 6400 ± 30 BP (Beta-398967); and here chronologically positioned between 7400 and 7201 cal BP.

Cingle de Mas Cremat (Portell de Morella, Castelló)

Archaeological work on this site situated in a rock settler began in 2006 to delimit the potential impact on cultural heritage arising from the installation of the infrastructure associated with the construction of a Wind Park [23]. The trench enabled the identification of a stratigraphic sequence comprising seven sedimentary units, encompassing archaeological assemblages from the Late Mesolithic (levels V and VI), a transition to the Early Neolithic (level III), and a final level attributed to the Late Neolithic (level II). From this sequence, we will include, in chronological order, 8 geometric artifacts from level **V** (L-4), one geometric artifact from level **VI** (L-4), and one microlith from level **III** (L-4) [23]. The first is situated in the chronological window of 7800-7601 cal BP, given the dating of 6800 ± 50 BP (Beta-232341). Level VI is also included in this time frame, with a date of 6750 ± 50 BP (Beta-232342), placing it in the subsequent timeframe as well (7600-7401 cal BP). Finally, level III, with a date of 5960 ± 50 BP (Beta-232340), is chronologically positioned between 7000 and 6801 cal BP.

Costamar (Ribera de Cabanes, Castellón)

The site is situated in a vast coastal open-air area. In its prehistoric phase, the site takes the form of a field of silos, wherein the archaeological structures are primarily negative features or silos, probably useful to serve as containers for material remains [24]. Specifically, here we consider to be included in the sample silos with Neolithic affiliation (Costamar **NII**) (L-4). A total of 12 geometric microliths have been included in the study, dating to the most recent temporal time-bin (7000-6801 cal BP), based on published dates of 5965 ± 25 BP (UCIAMS56073) and 5996 ± 38 BP (OxAV23578).

Cova de Cocina (Dos Aguas, Valencia)

Among the excavation efforts at Cueva de la Cocina, notable campaigns occurred in the 1940s under the direction of Pericot [25] and in 1974 under the management of Fortea [26–28]. Subsequently, from 2015 to 2018, excavations were resumed by a team led by Dra. Oreto García Puchol, Sarah McClure and Joaquim Juan Cabanilles. Consequently, a proposal for the division of the sequence into a total of eighteen cultural subphases emerged, with this study incorporating five Mesolithic subphases. In chronological order, geometric microliths [29] from the Cocina **A1** (57 pieces), **A2** (85 pieces), **B1** (96 pieces), **B2** (173 pieces), and **B3** (20 pieces) subphases are included (L-4).

Regarding their chronological association in this study, subphase **A1**, dated at 7610 ± 40 BP (Beta-267440) and 7415 ± 35 (UCIAMS-147347), has been placed within the chronological time-bins of 8600-8401 and 8400-8201 cal BP. Following A1 is subphase **A2**, which, with a date of 7475 ± 25 BP (UCIAMS-145195), falls within the same time-bin of 8400-8201 cal BP and extends into the subsequent timeframe of 8200-8001 cal BP. For Phase B, subphase **B1** is included in the chronological time-bins of 8000-7801 and 7800-7601 cal BP based on BP dates of 7050 ± 50 (Beta-267437), 6980 ± 30 (Beta-599656), 6970 ± 30 (Beta-599657), and 6940 ± 30 (Beta-512548). Subphase **B2** is dated at 6970 ± 35 (UCIAMS-147346), 7080 ± 50 (Beta-267436), 6760 ± 30 (Beta-599654), and 6880 ± 30 (Beta-599655), placing it within the same chronological time-bins. Finally, phase **B3** is also included in the latter time-bin of 7800-7601 cal BP, with BP dates of 6840 ± 50 (Beta-267435) and 6760 ± 30 (Beta-512549), and extends into the subsequent timeframe of 7600-7400 cal BP with dates of 6590 ± 25 (PSU5323) and 6705 ± 35 (UCIAMS-174945) BP.

Cova de l’Or (Beniarrés, Alicante)

Situated on the eastern flank of the Benicadell mountain range at an elevation of 650 meters above sea level, the cave has been the subject of excavation activities since the mid-20th century. The initial excavations, predominantly conducted by Vicent Pascual, yielded a substantial portion of the site's materials. Subsequent excavations carried out by Martí in the 1980s further contributed to the archaeological understanding of Cova de l’Or. The site's archaeological sequence encompasses various Neolithic phases, including the Early Cardial Neolithic (Neolithic IA) characterized by cardial ware, and the Early Cardial/Epicardial Neolithic (Neolithic IB), among other more recent episodes.

To optimize material retrieval, this study adheres to the periodization considered by Garcia-Puchol [30] and Juan-Cabanilles [31], focusing specifically on his Neolithic IB, IA and incorporating the earliest levels from the more recent excavations [32,33], not fully published. Within the scope of this work, 82 geometric artifacts from this site have been examined. Sampled by archaeological level, we sampled a total of 27 pieces belonging from Or **V** (L-4) with a dating of 6340 ± 40 BP (Beta298125) and 6356 ± 23 BP (MAMS19063) thus incorporated to 7400-7200 cal BP. The rest of the pieces are included in 7400-7200 as well as 7200-7001 cal BP samples, both for levels **IV** (11 pieces) and **VI** (44 pieces) (L-4). The level IV with C14 date for 6290 ± 70 (Beta298124), and the VI with 6310 ± 70 (OxA10192), 6275 ± 70 (OxA10191) and 6290 ± 75 (H1754-1208).

Cova de les Cendres (Teulada-Moraira, Alicante)

This open cavity situated in Teulada-Moraira, Alicante, represents an erstwhile karst formation within the coastal cliffs extending from Cap de la Nau to Puntal de Moraira. Archaeological investigations encompassed approximately 50 m^2^ of this cavity, revealing a substantial chronological sequence spanning the Paleolithic, Neolithic, and Bronze Age periods. The excavation of its Neolithic layers took place in the 1980s and 1990s [34], with renewed exploration initiated in 2018. This study incorporates microliths from several levels. In chronological order, level **XI** (L-4) contributes with 3 geometrics. This sedimentary level presents an unpublished C14 date of 6580 ± 30 (named temporary Cendres07) on cereal so that the first-time bin where it is included is 7600-7401 cal BP. Also, the unit is dated by 6468 ± 25 BP (ETH107002) and 6482 ± 25 BP (ETH107001), thus including chronological testimony for 7400-7201 cal BP. Other 2 microliths belong to level **IX** (L-4) in 7200-7001 cal BP with a C14 date of 6224 ± 45 (CNA483411) as well as 1 microlith from **VIIa** (L-4) in 7200-7001 and 7000-6801 cal BP according to the C14 date 6133 ± 37 BP (CNA483312). Additionally, one was recovered one geometric in the intermedial level **X** (L-4) inner 7400-7201 and 7200-7001 cal BP. However, because this geometric did not meet our appearance criteria (the paradigmatic type is not present in more than 1 context in the sample), the level has been discarded from the network analysis.

Coves del Fem (Uldemolins, Tarragona)

The site, discovered in 1997, is located in a rocky shelter. Excavation efforts conducted in 2013 and 2015 allowed for the documentation of a stratigraphy with a thickness of 140 cm, containing at least nine levels attributed to occupations from the late Mesolithic to the early Neolithic [35]. Specifically, the three lower units (UE 106-108, 107, and 109) are attributed to Mesolithic occupations, while the six upper units are associated with Neolithic occupations, featuring cardial (UE 102, 103-103a, 104) and epicardial assemblages (UE 2, 3, 2103, 2104). Here are included 4 geometric artifacts from the cardial level **103** (L-3) [36], chronologically situated in 7600-7400 cal BP based on the dating of 6570 ± 30 BP (Beta-42865). In this instance, the selected date corresponds to the context shared with the lithic assemblage sampled.

Cueva de Chaves (Casbas, Huesca)

Initial systematic excavations happened first in 1974/75 and later in 1984 and 1990. The denomination of the stratigraphic sequence was set in the latest campaigns, when a Palaeolithic phase and two Neolithic episodes were recognized, Level 1b attached to a cardial moment and Level 1a, occupied sometime during the recent cardial [37]. A total of 38 geometric artifacts from level **Ib** (L-4) and 6 microliths from level **Ia** (L-4) have been collected [37]. The first of these levels is chronologically situated in 7600-7401 and 7400-7201 cal BP, based on radiocarbon dates of 6580 ± 35 BP (GrA-38022), 6380 ± 40 BP (GrA-28341), and 6470 ± 25 BP (UCIAMS-66317). The second, on the other hand, is placed in the subsequent time-bin of 7200-7001 cal BP, given radiocarbon dates of 6227 ± 28 BP (MAMS-28127) and 6180 ± 54 BP (D-AMS015831).

Cueva de Nerja (Nerja, Málaga)

Situated within a spacious cave positioned at an elevation of 158 m.a.s.l., to the south of the Almijara Mountain range and approximately 1000 meters inland from the coast, this site has witnessed human occupation in its external rooms spanning from around 25000 to 4000 cal BP. The archaeological interventions conducted in this cave have been diverse and extended over time, involving comprehensive parietal surveys and systematic excavations in the external rooms named Mina, Vestíbulo, and Torca [38,39]. This occupancy encompasses both the Upper Pleistocene and Chalcolithic phases, as documented in the material record [40]. The materials employed in this investigation consisted of 6 geometric microliths sourced from distinct rooms [41], namely Vestíbulo (NV) and Mina (NM), and various levels, specifically **NV2** (1) and **NM10** (5) (L-4). These artifacts were recovered during Jordá's interventions spanning the years 1979 to 1987. The dating of 6342 ± 37 BP (OxA-26085) was utilized as a chronological reference for NM10 and has been included in the 7400-7201 cal BP sample. The geometric located in NV2 was dated with 6185 ± 21 BP (MAMS-20437) this is included in the 7200-7001 cal BP sample.

Cueva del Toro (Antequera, Málaga)

Positioned at an elevation of 1190 meters above sea level in El Torcal de Antequera, a karst mountain range confining the province of Málaga to the north, Toro cave has been the focus of five systematic excavation campaigns conducted in the years 1977, 1980, 1981, 1985, and 1983. These campaigns revealed a stratigraphic sequence that delineates human occupation from the mid-8th millennium to the early 4th millennium cal BP [42]. Notably, Phase **IV** (L-4), dated to 6200 ± 30 cal BP (Beta-365294) on the short-lived sample, corresponds to an Ancient Neolithic habitation phase characterized by the primary activity of meat product exploitation and subsequent treatment [42,43]. Within the scope of this study, four geometric microliths selected from Toro cave are examined [44] and included in a 7200-7001 cal BP sample.

El Abrigo de Valcervera (Biel, Zaragoza)

In 2004, a team led by Rafael Domingo and Lourdes Montes conducted the initial excavation campaign at the site [45]. This first campaign resulted in the documentation of three stratigraphic test trenches, with only one revealing stratigraphy containing levels of human occupation. Due to the scarcity of diagnostic materials found, partly attributed to the small size of the trench, excavation efforts were resumed in 2009. As a result of both campaigns, two archaeologically fertile units, labeled levels a and b, were documented. Among these, the second level (b) is the only one accepted as intact [46]. Thus, a total of 4 microliths documented in the **level b** (L-4) of the site are included in this study [47]. This assemblage would be temporally situated in the range of 8000-7801 cal BP based on dates of 6995 ± 40 BP (GrA-45783) and 7035 ± 45 BP (GrA-45763).

El Collado (Oliva, Valencia)

El Collado is an open-air site located in Oliva, Valencia. Archaeological work happened in the late 1980s [48] identified a shell-midden deposit with different phases of human occupation, as well as evidence of 14 burials dated to the Early Holocene [49]. The archaeological deposit, formed by colluvial and anthropic deposition and ranges between 1 and 1.5 m depth, was originally linked with three levels, I, II, and III, with Mesolithic chrono-cultural attributions [48]. Subsequently, an additional level (IV) was included in the sequence after a stratigraphic and assemblage revision under the direction of Fernández-López de Pablo. This study incorporates a total of 4 artifacts from the level **Collao I** (Phase 3) (L-4), situated in the chronological time-bin of 8600-8401 cal BP based on a dating of 7660 ± 44 BP (UBA-27478). It is noteworthy to mention that there is another short-lived dating (Beta-337186) for this same level (Layer C1 Quadrant F/III-GIII), which has been omitted due to its designation as inverted, as indicated by Fernández-López de Pablo [49].

El Esplugón (Sabiñánigo, Huesca)

In this archaeological site, fieldwork took place between the years 2009 and 2013. Six archaeological levels were documented, including various cultural phases from the Magdalenian to the Early Neolithic [50]. Due to the identification of various anomalies and discussions regarding the potential displacement of some available dates across different levels, this study only incorporates 47 geometric artefacts from level **3inf** (L-4). The Esplugón 3inf level is attributed to the Geometric Mesolithic and includes a dating of 6950 ± 50 (Beta-306723) that aligns with the documented cultural record. According to the dating, the sample fits within the chronological time-bins of 8000-7801 and 7800-7601 cal. BP.

Espantalobos (Quicena, Huesca)

The archaeological site, situated in the pre-Pyrenean basin within a sandstone shelter, was discovered in the 1980s and initially excavated in 2013 under the direction of Rafael Domingo and Lourdes Montes [51] Subsequent excavations took place in 2014 and 2015 [52]. Within its sequence, four sedimentological levels were identified, containing evidence of at least two episodes of human occupation during the Geometric Mesolithic period (levels c and e). However, only one of these, **level c** (L-4), has provided sufficient radiocarbon dates and archaeological material for the accurate assignment of the unit. Hence, this study includes a total of 7 geometric microliths [51]. Dated at 7390 ± 40 BP (Beta361624), the level has been placed within the chronological time-bins of 8400-8201 and 8200-8001 cal BP.

Forcas II (Graus, Huesca)

Excavation efforts at this archaeological site commenced in 1990 and continued through 1992, 1996, and 1997 [53]. It is noteworthy that the area s intervened in each campaign varied, along with the nomenclature of the stratigraphy. Among all the intervened areas, the western zone of the site retains the most robust stratigraphic profile, featuring a sequence spanning from the Mesolithic notched and denticulated artifacts (level Ib) to the Middle Neolithic. This sequence includes levels representing the Geometric Mesolithic (levels II and IV) and the transition to the Early Neolithic (levels V and VI). Of the entire documented lithic industry, this study incorporates 38 geometric microliths from the Forcas **II** (14 pieces) and **IV** (26 pieces) levels (L-4). Both levels are situated in the chronological time-bin of 8000-7801 cal BP based on dates obtained, namely 7150 ± 40 BP (Beta-250944) for Forcas II and 7000 ± 40 BP (Beta-290932) for Forcas IV.

La Draga (Banyoles, Barcelona)

The La Draga archaeological site currently exists partially on dry land and partially covered by the lake water table, and these conditions have contributed to the exceptional preservation of constructed structures, objects crafted from wood and vegetable fibers, and various other organic materials. The first excavations were carried out between 1991 and 2005 (Sectors A, B, and C), and the framework was resumed in 2010 (Sector D) [54]. These archaeological excavations have documented various structures that would correspond to an Early Neolithic settlement in which evidence of Cardial pottery has been identified [54,55].

According to a recent study made on radiometric, dendrochronological, and sedimentary data [56], the predominant chronological model suggests two main Neolithic occupations, further divided into at least three phases. These phases involve the construction, use, and repair of foundational wooden platforms, and later constructions are evident after the ground surface was reorganized using travertine slabs. However, contextual details for the microliths were not found. In this sense, the highest level of detail to which we could attribute the artifacts, in some instances, was the sector of their recovery. Consequently, the 14 artefacts [55] are associated with the entirety of the published short-lived life radiocarbon dates on cereal for the site (see Table 1 in the main text) (L-4), which are the dates we select to incorporate the site in a chronological time frame. Thus, geometric are included in the time bins 7400-7201, 7200-7001, and 7000-6801 cal BP.

Les Guixeres de Vilobí (Sant Martí Sarroca, Barcelona)

Systematic excavations in this open-air-site conducted initially from 1974 to 1984 and later resumed in 2015 revealed a stratigraphic sequence encompassing three distinct occupational phases: Phase A denoting the Ancient Cardial Neolithic level, Phase B representing the Ancient Epicardial Neolithic level, and Phase C signifying the Cardial Post-Neolithic level [57,58]. Within the Ancient Cardial Neolithic phase, specifically in Ámbito 1 and Ámbito 2, two habitation areas were meticulously documented, featuring numerous negative structures such as hole posts and silos [59]. Utilizing the site's oldest date on *Ovis aries*, recorded as 6655 ± 45 (OxA-26068), this stands as one of the earliest dates for the Iberian Peninsula. For this study, 5 geometric microliths from the Ancient Cardial level (**Phase A**) (L-4) were employed, with four sourced from Mestres [57] and the remaining one from Gibaja et al. [60]. Due to the date, the pieces are part of the sample of 7600-7401 cal BP. Also, we have employed one microlith from **Phase B** (L-4) [57], which is included in the time bin 7000-6801 cal BP due to the C14 date 5980 ± 30 (Beta574950).

Mas d’Is (Penàguila, Alicante)

Situated within an ancient glacis amid two ravines in Penàguila, Alicante, this open-air site has been the subject of numerous excavation campaigns since the mid-1990s. Noteworthy discoveries include various residential structures at the site's core, including identified huts. Dating back to the Ancient Neolithic level (**VIb**) (L-4), a specific date of 6600 ± 50 BP (Beta162092) has been associated with *Hordeum sp*. [61]. Additionally, the site has yielded *Impressa* decorated ware. In this case, we have used 2 microliths belonging to this level in the timeframe of 7600-7401 cal BP [62,63].

Mas Nou (Ares del Maestre, Castellón)

The site, discovered in 1975, is an open-air site at 940 masl. It was excavated in 1987/88 and between 1999 and 2011 [64]. The stratigraphic sequence includes five levels where were identified occupation events for Geometric Mesolithic (III and IV) and Neolithic (I and II) periods. Here, we select the closed context of a burial from level III (L-1) which includes a total of two geometric [64] linked to radiocarbon dates of 6925 ± 35 (OxA-V-2360-29) and 6897 ± 34 BP (OxA-V-2360-28) both made on human bone [65]. Thus, the sample is included in 7800-7601 cal BP time frame.

Pontet (Maella, Zaragoza)

The archaeological site, located in a shallow shelter, was cataloged in 1984 and 1985 by a team from the University of Zaragoza led by P. Utrilla. Subsequently, excavation interventions between 1986 and 1989 happened under the direction of C. Mazo and L. Montes [66]. The excavation efforts documented a total of ten stratigraphic levels, beginning with a sterile level (level j), upon which various occupation events occurred. These events span from the Mesolithic notched and denticulated artifacts (levels i and g) to the Neolithic (upper levels c and b), including the Geometric Mesolithic (level e) as well as some sterile layers (levels h, f, d) and transitional layers (lower-level c). In this instance, a total of 14 geometric artifacts from **level e** (L-4) are included, dating to 8200-8001 cal. BP based on available dates: 7341 ± 32 BP (DAMS-020210) and 7340 ± 70 (GrN-16313). Due to the scarcity of faunal remains at the site, all available dates are derived from charcoal samples. In this study, we choose to reserve the upper-level c, originally attributed to the Early Neolithic, considering the coexistence in the record of materials characteristic of non-ceramic groups (such as microburins) and the antiquity of the available dating (D-AMS 020208).

Els Trocs cave (Bisaurri, Huesca)

Els Trocs cave is located in the southern Pyrenees. In the site, consists of a single chamber, archaeological works uncovered a total area of 45 m^2^ during seven fieldwork seasons (2009-2012. 2014, 2016 and 2019) under Manuel Rojo-Guerra and José I. Royo-Guillén [67]. The stratigraphic sequence includes four occupation phases dated to the Neolithic (Trocs I-III) and to the Roman and modern periods (Trocs IV). Regarding the Neolithic layers, Trocs I comprises the main archaeological findings with several structures (pits of different sizes) and a floor intentionally fashioned from 17000 decorated potsherds fragments [67]. Along with combustion traces, and a large quantities of archaeological remains, in the cave archaeologists found human skeletal of at least nine individuals with violence traces [68]. Findings are poorest in Trocs II, a level interpretate as a floor regularization event associated with a large hearth and two dense accumulations of charred material which do not correspond with a *fumier* event [67]. Here, we incorporate a total of two geometric points [69] located in **UE 53** (L-1) of phase Trocs I. With a radiocarbon date of 6080 ± 40 BP (Beta-316512), UE 53 is located in the 7000-6801 cal BP time frame.

Valmayor (Mequinenza, Zaragoza)

Valmayor site, situated on a sheltering rock at an elevation of 120 meters above sea level, strategically occupies a position between the Ebro and Matarraña valleys within a sandstone and water catchment region. While the site gained recognition in the 1980s through rock art studies [70], the comprehensive archaeological sequence emerged from systematic excavations initiated in 2011 [71]. Recent investigations have identified three distinct occupation phases occurring at various points during the VIII millennium cal BP: Valmayor XI-I, XI-II, and XI-III. Notably, there is a phase of population gap between the latter phases. This study includes a total of 5 geometrics located in the Late Neolithic level **XI-III** (L-4) [71], excluding pieces from the previous level XI-II to avoid the possible mixing of cultural material which guided researchers to understand the site as a Mesolithic site with the influence of nearby Neolithic settlements [71]. The sample is located in the last time bin, 7000-6801 cal BP, due to the available radiocarbon date in 6090 ± 30 BP (Beta-341167).

# REFERENCES

1. García-Martínez de Lagrán I, Iriarte E, García-Gazólaz J, Tejedor-Rodríguez C, Gibaja-Bao JF, Moreno-García M, et al. 8.2 ka BP paleoclimatic event and the Ebro Valley Mesolithic groups: Preliminary data from Artusia rock shelter (Unzué, Navarra, Spain). Quat Int. 2016;403: 151–173. doi:10.1016/j.quaint.2015.06.050

2. García-Puchol O, Aura-Tortosa JE. El abric de la Falguera (Alcoi, Alacant): 8.000 años de ocupación humana en la cabecera del río de Alcoi. Ayuntamiento de Alcoy. Ayuntamiento de Alcoy; 2006.

3. Oms FX, Sánchez de la Torre M, Petit MÀ, López-Cachero FJ, Mangado X. Nuevos datos del VI y V milenio cal BC en el llano y Prepirineo de Lleida (NE de la Península Ibérica): el Abric del Xicotó y Les Auvelles. Munibe Antropol-Arkeol. 2019; 93–107. doi:doi.org/10.21630/maa.2019.70.05

4. Domingo-Martínez R, Bea-Martínez M, Utrilla-Miranda, María del Pilar. Una nueva ocupación neolítica en el río Guadalope: la campaña de 2009 en el abrigo de Ángel 2. Salduie Estud Prehist Arqueol. 2010; 225–236.

5. Utrilla Miranda P, Domingo R, Bea-Martínez M, editors. El Arenal de Fonseca (Castellote, teruel): Ocupaciones preshistóricas del Gravetiense al Neolítico. Zaragoza: Prensas de la Universidad de Zaragoza; 2017.

6. Soto A. Producción y gestión de la industria lítica de Atxoste (Álava): Una aproximación a las sociedades Epipaleolíticas y Mesolíticas del alto Ebro. Universidad del País Vasco - Euskal Herriko Unibertsitatea. 2014.

7. Pérez-Fernández AJ, Ligouis B, Alday A. Reconstructing climate and soil formation processes in the Atxoste rock-shelter (Upper Ebro Valley, Northern Spain): A preliminary geoarchaeological view of human communities during the Pleistocene-Holocene transition in the Ebro Valley and Cantabrian Spain. J Archaeol Sci Rep. 2020;31: 102286. doi:10.1016/j.jasrep.2020.102286

8. Soto A, Rodríguez-Lejarza A, Jiménez L, Domingo R, Utrilla P, Montes L, et al. There and back again: Late Mesolithic technological change in the northeast of the Iberian Peninsula. J Archaeol Sci Rep. 2023;50: 104086. doi:10.1016/j.jasrep.2023.104086

9. Esquembre Bebiá MA, Boronat Soler J de D, Jover Maestre FJ, Molina Hernández FJ, Luján Navas A, Fernández López de Pablo J, et al. El yacimiento neolítico del Barranquet de Oliva (Valencia). IV Congreso del Neolítico Peninsular. 2008. pp. 183–190.

10. Torregrosa-Giménez P, Jover-Maestre FJ, López-Seguí E. Benàmer (Muro d’Alcoi, Alicante): mesolíticos y neolíticos en las tierras meridionales valencianas. Servicio de Investigación Prehistórica del Museo de Prehistoria de Valencia. Valencia: Diputación de Valencia; 2011.

11. Barandiarán I. El abrigo de la Botiqueria dels Moros. Mazaleón (Teruel). Excavaciones arqueológicas de 1974. Quad Prehistòria Arqueol Castelló. 1978; 49–138.

12. Rodanés JM, Picazo JV, editors. El campamento mesolítico del Cabezo de la Cruz La Muela. Zaragoza. Zaragoza: Prensas de la Universidad de Zaragoza; 2013.

13. Fernández-López de Pablo J, Salazar-García DC, Subirà-Galdacano ME, Roca de Togores C, Gómez-Puche M, Richards MP, et al. Late Mesolithic burials at Casa Corona (Villena, Spain): direct radiocarbon and palaeodietary evidence of the last forager populations in Eastern Iberia. J Archaeol Sci. 2013;40: 671–680. doi:10.1016/j.jas.2012.09.005

14. Molist M, Vicente-Campos O, Farré i Barrufet R. El jaciment de la caserna de Sant Pau del Camp: aproximació a la caracterizació d’un asentament del neolític antic. Quarhis Quad Arqueol Història Ciutat Barc. 2008; 14–24.

15. Borrell F, Gibaja-Bao JF. The First Neolithic Communities in Northeast Iberia: Procurement, Production, and Use of Lithic Tools at the Settlement of Caserna de Sant Pau Del Camp (Barcelona, Spain). J Isl Coast Archaeol. 2012;7: 313–337. doi:10.1080/15564894.2011.652764

16. Molist-Montaña M, Gómez-Bach A. La Caserna de Sant Pau del Camp y la caracterización de los conjuntos de cerámica impresa con malacofauna dentada del noreste de la península Ibérica. In: Pardo-Gordó S, Gómez-Bach A, Bernabeu-Auban J, editors. Contextualizando la cerámica impressa: Horizontes culturales en la península ibérica. Barcelona: Bellaterra : Universitat Autònoma de Barcelona. Servei de Publicacions; 2020. pp. 89–100.

17. Arribas A, Molina F. El poblado de “los Castillejos” en las peñas de los gitanos (Montefrío, Granada): campaña de excavaciones de 1971: el corte no. 1. Granada: Secretariado de Publicaciones para el Departamento de Prehistoria de la Universidad de Granada; 1979.

18. Cámara Serrano JA, Molina González F, Afonso Marrero JA. La cronología absoluta de Los Castillejos en Las Peñas de los Gitanos (Montefrío, Granada). Cantabria: Universidad de Cantabria; 2005.

19. Martínez-Fernández G, Afonso-Marrero JA, Cámara-Serrano JA, Molina-González F. Contextualización cronológica y análisis tecnotipológico de los artefactos tallados del Neolítico antiguo de Los Castillejos (Montefrío, Granada). In: Gibaja-Bao JF, Faustino-Carvalho A, editors. Os últimos caçadores-recolectores e as primeiras comunidades productoras do sul da Península Ibérica e do norte de Marrocos. Faro: Promontoria Monográfica; 2010. pp. 163–171.

20. Molina González F, Cámara Serrano JA, Alfonso Marrero JA, Gámiz Caro J, Capel Martínez J, Martínez Fernández G. Hiatus in an archaeological multilevel site: Los Castillejos in Las Peñas de los Gitanos (Montefrío, Granada). Beyond limits Studi in onore di Giovanni Leonardi. Università degli Studi di Padova; 2017. pp. 91–100.

21. Blasco A, Edo M, Villalba MJ, Saña M. Primeros datos sobre la utilización sepulcral de la Cueva de Can Sadurní (Begues, Baix Llobregat) en el Neolítico Cardial. III Congreso del Neolítico en la Península Ibérica. Santander: Universidad de Cantabria Servicio de Publicaciones; 2005. pp. 625–634.

22. Blasco A, Edo M, Villalba J, Buxó R, Juan-Tresserras J, Saña M. Del cardial al postcardial en la cueva de Can Sadurní (Begues, Barcelona). Primeros datos sobre su secuencia estratigráfica, paleoeconómica y ambiental. II Congrés del Neolític a la Península Ibèrica. Saguntum: Papeles del Laboratorio de Arqueología de Valencia; 1999. pp. 59–67.

23. Vizcaíno-León D, editor. El Cingle del Mas Cremat (Portell de Morella, Castellón). Un asentamiento en altura con ocupaciones del Mesolítico Reciente al Neolítico Final. Parque Eólico de las Cabrillas. Zona III del Plan Eólico Valenciano. Renomar SA, EIN Mediterráneo SL. Valencia: Generalitat Valenciana; 2010.

24. Flors E, editor. Torre la Sal (Ribera de Cabanes, Castellón). Evolución del paisaje antrópico desde la prehistoria hasta el medievo. Castellón: Servei d’Investigacions Arqueològiques i Prehistòriques; 2009.

25. Pericot L. La cueva de la Cocina (Dos Aguas). Nota preliminar. Arch Prehist Levantina. 1946;II: 39–71.

26. Fortea J. La Cueva de la Cocina. Ensayo de cronología del Epipaleolítico (Facies Geométricas). Trabajos Varios del SIP. Valencia: Diputación de Valencia; 1971.

27. Fortea J. Los complejos microlaminares y geométricos del Epipaleolítico mediteráneo español. Salamanca: Universidad de Salamanca; 1973.

28. Fortea Pérez J, Martí Oliver B, Fumanal García MP, Dupre Ollivier M, Pérez Ripoll M. Epipaleolítico y neolitización en la zona oriental de la Península Ibérica. Actes du Colloque Premières communautés paysannes en Méditerranée occidentale. Montpellier, París.: CNRS Éditions-Histoire; 1987. pp. 581–591. doi:10.4000/books.editionscnrs.1148.

29. Cortell-Nicolau A, García-Puchol O, Cabanilles JJ. The geometric microliths of cueva de la cocina and their significance in the mesolithic of Eastern Iberia: A morphometric study - ScienceDirect. Quat Int. 2023; xxxx. doi:https://doi.org/10.1016/j.quaint.2023.05.014

30. García-Puchol O. El proceso de neolitización en la fachada mediterránea de la península ibérica. Tecnología y tipología de la piedra tallada. Oxford: BAR International Series; 2005.

31. Juan Cabanilles J. El utillaje de piedra tallada en la Prehistoria reciente valenciana: aspectos tipológicos, estilísticos y evolutivos. SIP, Diputación de Valencia; 2008.

32. Martí Oliver B, Pascual Pérez V, Gallart Martí MD, Pérez Ripoll M, Acuña Hernández JD, Robles Cuenca F. Cova de l’Or (Beniarrés, Alicante). Vol. II. Valencia: Diputación de Valencia; 1980.

33. Martí Oliver B. La Cova de l’Or (Beniarrés, Alicante). Saguntum Papeles Lab Arqueol Valencia. 2011; 183–186.

34. Bernabeu-Auban J, Molina-Balaguer L, editors. La Cova de les Cendres. Alicante: MARQ. Museo Arqueológico Provincial de Alicante; 2009.

35. Bogdanovic I, Palomo A, Piqué R, Rosillo R, Terradas X. Los Últimos Cazadores-Recolectores en el NE de la Península Ibérica: Evidencias de Ocupaciones Humanas Durante el VI Milenio Cal BC. In: Barceló JA, Bogdanovic I, Morell B, editors. Actas del Congreso de Cronometrías Para la Historia de la Península Ibérica (IberCrono 2017). Barcelona: Universitat Autònoma de Barcelona; 2017. pp. 35–45.

36. Palomo A, Terradas-Batlle X, Piqué R, Rosillo R, Bodganovic I, Bosch À, et al. Les Coves del Fem (Ulldemolins, Catalunya). 2018.

37. Cava Almuzara A. La industria lítica del neolítico en Chaves, Huesca. Saldvie Estud Prehist Arqueol. 2000; 77–164.

38. Simón Vallejo MD. Una secuencia con mucha prehistoria: la Cueva de Nerja. Mainake. 2003; 249–274.

39. Gibaja Bao JF, Cortés Sánchez M, Simón Vallejo MD. La función del utillaje lítico neolítico. El ejemplo de la Cueva de Nerja (Málaga). SPAL - Rev Prehist Arqueol. 2010;19: 97–110. doi:10.12795/spal.2010.i19.04

40. Aura-Tortosa JE, Badal E, García-Borja P, Garcia-Puchol O, Pascual-Benito JL, Jordá-Pardo JF. Cueva de Nerja (Málaga). Los niveles neolíticos de la Sala del Vestíbulo. III Congreso de Neolítico en la Península Ibérica. Santander; 2005. pp. 975–987.

41. Aura Tortosa JE, Jordá Pardo JF, García Borja P, García Puchol O, Badal E, Pérez Ripoll M, et al. Una perspectiva mediterránea sobre el proceso de neolitización: los datos de la cueva de Nerja en el contexto de Andalucía (España). Menga Rev Prehist Andal. 2013; 53–78.

42. Martín Socas D, Massieu M, Gonzalez Quintero P. La Cueva de El Toro (Sierra de El Torcal-Antequera-Málaga). Un modelo de Ocupación Ganadera en el Territorio Andaluz entre el VI y II Milenios A.N.E. Consejería de Cultura. Junta de Andalucía. Sevilla; 2004.

43. Camalich Massieu MD, Martín Socas D. Los inicios del Neolítico en Andalucía. Entre la tradición y la innovación. MENGA Rev Prehist Andal. 2013;04: 103–129.

44. Rodríguez Rodríguez A, Gibaja JF, Perales Barrón U, Clemente Conte I. Comunidades campesinas, pastoras y artesanas. Traceología de los procesos de trabajo durante el Neolítico andaluz. MENGA Rev Prehist Andal. 2013;4: 35–52.

45. Montes L. El abrigo de Legunova en Biel: campaña de 2003. Saldvie. 2004; 395–406.

46. Domingo R, Montes L. Valcervera y Rambla de Legunova: dos yacimientos postpaleolíticos en Biel, Zaragoza. Saldvie. 2009; 295–310.

47. Montes L, Domingo R, González-Sampériz P, Sebastián M, Aranbarri J, Castaños P, et al. Landscape, resources and people during the Mesolithic and Neolithic times in NE Iberia: The Arba de Biel Basin. Quat Int. 2016;403: 133–150. doi:10.1016/j.quaint.2015.05.041

48. Aparicio J. La necrópolis mesolítica de El Collado (Oliva-Valencia). Valencia: Diputación provincial de Valencia; 2008.

49. Fernández-López de Pablo J. The timing of postglacial coastal adaptations in Eastern Iberia: A Bayesian chronological model for the El Collado shell midden (Oliva, Valencia, Spain). Quat Int. 2016;407: 94–105. doi:10.1016/j.quaint.2015.10.077

50. Utrilla-Miranda P, Berdejo-Arceiz A, Obón-Zúñiga A. El Esplugón: un gran abrigo mesolítico en el valle del Guarga (Huesca). In: Muñiz-Alvarez J, editor. Ad Orientem: del final del Paleolítico en el Norte de España a las primeras civilizaciones del Oriente Próximo : estudios en homenaje a Juan Antonio Fernández-Tresguerres Velasco. Ménsula; 2012. pp. 235–252.

51. Montes L, Domingo-Martínez R, Cuchí JA, Alcolea M, Sola C. Completando el mapa de la Cuenca del Ebro: el Mesolítico del IX milenio cal BP de Espantalobos (Huesca, España). Munibe Antropol-Arkeol. 2015; 119–133. doi:doi: 10.21630/maa.2015.66.06

52. Alcolea M, Domingo R, Piqué R, Montes L. Landscape and firewood at Espantalobos Mesolithic site (Huesca, Spain). First results. Quat Int. 2017;457: 198–210. doi:10.1016/j.quaint.2016.10.007

53. Utrilla P, Mazo C, editors. La Peña de las Forcas (Graus, Huesca). Un asentamiento estratégico en la confluencia del Ésera y el Isábena. Zaragoza: Monografías Arqueológicas. Universidad de Zaragoza.; 2014.

54. Bosch A, Chinchilla J, Tarrús J, editors. El poblat lacustre del neolític antic de La Draga. Excavacions 2000-2005. Girona: CASC. Museu d’Arqueologia de Catalunya; 2011.

55. Bosch A. El poblat lacustre neolític de la Draga: excavacions de 1990 a 1998 (Monografies del CASC 2). Girona: Centre d’Arqueologia Subaquàtica de Catalunya, Museu d’Arqueologia de Catalunya; 2000.

56. Andreaki V, Barceló JA, Antolín F, Gassmann P, Hajdas I, López-Bultó O, et al. Absolute chronology at the waterlogged site of La Draga (lake Banyoles, NE Iberia): Bayesian chronological models integrating tree-ring measurement, radiocarbon dates and micro-stratigraphical data. Radiocarbon. 2022;64: 907–948. doi:10.1017/RDC.2022.56

57. Mestres J. La indústria lítica en sílex del Neolític Antic de les Guixeres de Vilobí. Olerdulae Rev Mus Vilafranca. 1987; 3.

58. Mestres J. Avançament a l’estudi del jaciment de Les Guixeres de Vilobí. Pyrenae. 1981;17: 35–53.

59. Oms FX, Mestres J, Martínez-Grau H, Laborda R, Antolín F, Bergadà MM, et al. Fases de ocupación y estratigrafía del asentamiento neolítico de Les Guixeres de Vilobí (Sant Martí Sarroca, Barcelona). Trab Prehist. 2021;78: 257–276. doi:10.3989/tp.2021.12275

60. Gibaja-Bao JF, Oms FX, Mestres J, Mazzucco N, Palomo A. Primeros resultados sobre la función del utillaje lítico de las primeras comunidades neolíticas asentadas en les Guixeres de Vilobí (Sant Martí Sarroca, Barcelona). Sagvntvm Papeles Laboratiorio Arqueol Valencia. 2018;50: 35–56. doi:http://dx.doi.org/10.7203/SAGVNTVM.50.11603

61. Bernabeu Auban J, Orozco Kóhler T, Diez Castillo A, Gómez-Puche M, Molina Hernández FJ. Mas d’Is (Penàguila, Alicante): aldeas y recintos monumentales del Neolítico Inicial en el valle del Serpis. Trab Prehist. 2003;60: 39–59. doi:10.3989/tp.2003.v60.i2.80

62. Cortell-Nicolau A, García-Puchol O. Apuntes sobre la industria lítica del Mas d’Is en el contexto del Horizonte “Impressa” del Mediterráneo Occidental. In: Pardo-Gordó S, Gómez-Bach A, Molist Montaña M, Bernabeu Auban J, editors. Contextualizando la cerámica impressa: Horizontes culturales en la Península Ibérica. Barcelona: Universitat de Barcelona.; 2020. pp. 121–133.

63. Garcia-Puchol O, Gibaja-Bao JF, Bernabeu-Auban J, Orozco-Köhler T. Tecno-tipología y funcionalidad de los utensilios líticos tallados en las primeras ocupaciones del Neolítico Antiguo de Mas d’Is (Penàguila, Alicante). In: Bicho NF, Faustino-Carvalho A, editors. História, Teoria e Método da Arqueologia Actas do IV congresso de arqueologia peninsular. Faro: Promontoria Monográfica; 2011. pp. 59–71.

64. Olària-i-Puyoles C, editor. CINGLE DEL MAS NOU: VIDA Y MUERTE EN EL 7000 BP. Un campamento temporal del Mesolítico reciente, inmerso en los procesos de neolitización, con inhumación colectiva. MONOGRAFIES DE PREHISTÒRIA I ARQUEOLOGIA CASTELLONENQUES. Castellón: Servei d’Investigacions Arqueològiques i Prehistòriques; 2020.

65. Salazar-García DC, Aura JE, Olària CR, Talamo S, Morales JV, Richards MP. Isotope evidence for the use of marine resources in the Eastern Iberian Mesolithic. J Archaeol Sci. 2014;42: 231–240. doi:10.1016/j.jas.2013.11.006

66. Mazo C, Montes L. La transición Epipaleolítico-Neolítico Antiguo en el abrigo de El Pontet (Maella, Zaragoza). In: Utrilla P, editor. Aragón/Litoral Mediterráneo Intercambios culturales durante la Prehistoria. Zaragoza: Institución Fernando el Católico; 1992. pp. 243–254.

67. Tejedor-Rodríguez C, Moreno-García M, Tornero C, Hoffmann A, Lagrán ÍG-M de, Arcusa-Magallón H, et al. Investigating Neolithic caprine husbandry in the Central Pyrenees: Insights from a multi-proxy study at Els Trocs cave (Bisaurri, Spain). PLOS ONE. 2021;16: e0244139. doi:10.1371/journal.pone.0244139

68. Alt KW, Tejedor Rodríguez C, Nicklisch N, Roth D, Szécsényi Nagy A, Knipper C, et al. A massacre of early Neolithic farmers in the high Pyrenees at Els Trocs, Spain. Sci Rep. 2020;10: 2131. doi:10.1038/s41598-020-58483-9

69. Rojo-Guerra M, Peña-Chocarro L, Royo JI, Tejedor-Rodríguez C, García Martínez de Lagrán Í, Arcusa H, et al. Pastores trashumantes del Neolítico antiguo en un entorno de alta montaña: secuencia crono-cultural de la Cova de Els Trocs (San Feliú de Veri, Huesca). BSAA Arqueol. 2013.

70. Royo Guillén JI. El abrigo con grabados rupestres esquemáticos de “Valmayor” (Mequinenza, Zaragoza). I Congreso Internacional de arte rupestre. Caspe, Zaragoza: Bajo Aragon. Prehistoria.; 1986. pp. 179–190.

71. Rojo Guerra MA, Tejedor Rodríguez C, Jiménez Jiménez I, Peña Chocarro L, Royo Guillén JI, García-Martínez de Lagrán Í, et al. Releyendo el fenómeno de la neolitización en el Bajo Aragón a la luz de la excavación del Cingle de Valmayor XI (Mequinenza, Zaragoza). Zephyrus Rev Prehist Arqueol. 2015;LXXV: 41–71. doi:10.14201/zephyrus2015754171
